# BMDx2: A Tool for Integrating Toxicogenomics‐Based Dose‐Dependency Analysis and AOP‐Based Mechanistic Insights

**DOI:** 10.1002/smtd.202501728

**Published:** 2025-11-12

**Authors:** Angela Serra, Michele Fratello, Giorgia Migliaccio, Emanuele Di Lieto, Marcella Torres Maia, Laura Aliisa Saarimäki, Alisa Pavel, Alexandra Schaffert, Andreas Tsoumanis, Antreas Afantitis, Jack Morikka, Giusy del Giudice, Dario Greco

**Affiliations:** ^1^ Finnish Hub for Development and Validation of Integrated Approaches (FHAIVE) Faculty of Medicine and Health Technology Tampere University Tampere 33100 Finland; ^2^ Division of Pharmaceutical Biosciences Faculty of Pharmacy University of Helsinki Helsinki 00790 Finland; ^3^ Department of Applied Mathematics and Computer Science Technical University of Denmark Kongens Lyngby 2800 Denmark; ^4^ Entelos Institute Larnaca 6059 Cyprus; ^5^ NovaMechanics Ltd Nicosia 1070 Cyprus; ^6^ NovaMechanics MIKE Piraeus 18545 Greece

**Keywords:** adverse outcome pathways, benchmark dose analysis, point of departure, toxicogenomics

## Abstract

Despite the advent of mechanistic toxicology using omics data to link molecular perturbations with systemic outcomes, regulatory toxicology still lacks the application of mechanism‐anchored metrics from such data. This is partially because traditional gene‐centric analysis often falls short of linking molecular changes to adverse outcomes. To address this gap, BMDx2, an open‐source tool that transforms multi‐dose toxicogenomics datasets into quantitative, mechanistic evidence for human chemical safety assessment is developed. BMDx2 couples benchmark‐dose modeling with Adverse Outcome Pathway (AOP) enrichment to derive transcriptomic‐based points of departure, enabling potency ranking, chemical prioritization, and mechanistically anchored explanations of the effect of chemical exposures. BMDx2 can process a broad range of data, including DNA microarray and RNA sequencing studies. Here, case studies are used to illustrate the versatility of BMDx2 in characterizing the mechanism of action of chemicals. An initial case study on carbon nanotubes exposure applies integrative analysis of transcriptomics and genome‐wide DNA methylation data, uncovering cellular reprogramming processes underlying fibrosis. A second case study on bleomycin exposure demonstrate how transcriptomic data alone can be mapped to fibrosis‐related AOPs in a standardized, regulatory appropriate manner. Together, these examples show how BMDx2 supports the regulatory application of toxicogenomics and accelerates mechanism‐based chemical safety evaluation.

## Graphical abstract

BMDx2 is an open‐source tool that integrates benchmark‐dose modeling with Adverse Outcome Pathway (AOP) enrichment to convert omics data into quantitative, mechanistic evidence. Case studies on rigid multi‐walled carbon nanotubes and bleomycin demonstrate its ability to reveal fibrosis‐related mechanisms, supporting both mechanistic discovery and regulatory application in next‐generation chemical safety assessment.

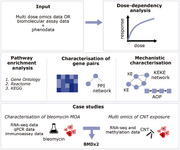

## 1 Introduction

Modern risk assessment increasingly emphasizes mechanistic understanding while reducing animal testing, as reflected in the shift toward Next Generation Risk Assessment (NGRA).^[^ ^1^, ^2^ ^]^ In this context, toxicogenomics, which provides data‐rich mechanistic insights into cellular responses, has emerged as a transformative approach.^[^ ^3^, ^4^, ^5^ ^]^

Traditionally, toxicogenomics has described the Mechanism of Action (MoA) as lists of deregulated molecules (e.g., genes, transcripts or proteins) in response to the exposure.^[^ ^3^, ^4^, ^5^ ^]^ However, this gene‐centric approach does not capture the complexity of the multiscale interactions between a chemical and the exposed biological system, and often fails to describe how molecular alterations lead to the observed toxicological effect in a mechanistic fashion. From a systems toxicology perspective, toxicological responses arise from the coordinated effect of numerous molecular events in the biological system under study.^[^ ^6^ ^]^ Such coordinated responses are often explained by multiple interactions happening at various levels, such as cell‐cell communication, protein‐protein interaction (PPI) or harmonized transcription regulation by transcription factors. The described MoA should be elevated from individual gene signatures to functional level perturbations, which then need be contextualized within a broader framework of biological organization. For example, the integration of diverse layers of molecular alteration through multi‐omics analysis, can better represent and predict the response of the exposed system.^[^ ^7^ ^]^ However, interpreting the MoA as a multi‐layer mechanism is challenging especially in the absence of prior knowledge or manual description of the observed response, and there is currently a lack of methods that link molecular evidence with toxicologically relevant events in a generalized manner. These interpretation challenges are currently limiting the regulatory application of toxicogenomics, specifically the derivation of decision‐relevant limit values and the functional contextualization of the molecular responses within regulatory‐relevant frameworks.^[^ ^8^ ^]^ Such frameworks include the Adverse Outcome Pathway (AOP),^[^ ^9^ ^]^ which provides a standardized approach to functionally and causally relate molecular perturbations to outcomes of regulatory concern. An AOP links a molecular initiating event (MIE) via causally supported key events (KEs) and key event relationships (KERs) to an adverse outcome at higher levels of biological organization. We previously showed that by mapping transcriptomic responses to KEs and AOPs, dose‐response signals can be translated into mechanism‐based, decision‐relevant limit values, thereby improving interpretability and regulatory applicability.^[^ ^10^, ^11^, ^12^ ^]^ In this context, applying benchmark‐dose (BMD) modeling to transcriptomic data yields gene‐level transcriptomic points of departure (tPODs),^[^ ^11^, ^13^ ^]^ which can be aggregated at KE/AOP levels for regulatory interpretation.^[^ ^14^, ^15^, ^16^ ^]^ Thus, a comprehensive workflow, that allows for consistent identification of dose‐responsive molecular alternations, and their integration into AOP contexts, supports the derivation of dose‐dependent MoA (ddMoA) and regulatory uptake of toxicogenomics data.

BMDx2 addresses this need by integrating dose‐response modeling with AOP‐centric mechanistic interpretation in a single, transparent workflow. BMDx2 is available as a Shiny software application and integrates three R packages: BMDx for dose–response analysis,^[^ ^13^ ^]^ AOPFingerprintR^[^ ^10^ ^]^ for the enrichment of KEs and AOPs, and FunmappOne^[^ ^17^ ^]^ for canonical pathway enrichment analysis. The BMDx and AOPFingerprintR packages are also offered as REST APIs. The platform (i) identifies dose‐responsive genes across DNA microarray, RNA‐seq, and other molecular data; (ii) derives gene‐level BMDs using multiple models with consensus averaging; (iii) aggregates to KE‐ and AOP‐level tPODs; (iv) supports correlation‐based exploration to compare tPODs within and across omics layers; (v) records parameters and decisions for full traceability. Outputs can be organized by human‐health hazard domains to aid communication and alignment with regulatory and mechanistically oriented Safe and Sustainable by Design (SSbD) discussions. In doing so, BMDx2 provides a cohesive platform to translate omics measurements into mechanism‐anchored, decision‐relevant metrics that are interpretable within emerging chemical risk assessment frameworks.

## 2 Experimental Section

### 2.1 Implementation and Data Analysis Pipeline

BMDx2 was an R‐Shiny application that integrates three key R packages (BMDx, FunMappOne, and AOPFingerprintR) into a unified environment for toxicogenomic dose‐response analysis (Figure S1, Supporting Information). Together, these packages enable the user to progress seamlessly through the full analytical workflow: from benchmark dose modeling (BMDx) to pathway enrichment (FunMappOne) and AOP based mechanistic annotation (AOPFingerprintR). While the BMDx2 interface streamlines this process, the individual R packages remain available for users who want to customize their workflow. In addition, BMDx and AOPFingerprintR functionalities were also available as REST APIs allowing integration into automated or large‐scale analysis pipelines (See section “*Implementation*” of the Supporting Information for more details). The dose‐dependent analysis functionalities offered by BMDx were structured into an established pipeline (Figure S2, Supporting Information). It begins with the input of an experimental toxicogenomics dataset and an accompanying metadata table describing the experimental conditions. An initial filtering step identifies features likely to exhibit dose‐dependent responses, using statistical approaches such as ANOVA, trend tests, or a *limma* based differential expression analysis. These pre‐selected features were then subjected to BMD modeling, where multiple mathematical models (Table S1, Supporting Information) were fitted to each profile across doses. Defining a benchmark response (BMR) was a key step, as it establishes what constitutes a biologically significant change, often set as a percentage above baseline. BMDx2 supports three approaches for determining BMR: relative, absolute, and standard deviation–based (Figure S3, Supporting Information). The selected BMR was mapped onto the fitted dose–response curve, and the dose corresponding to the predicted response shift (BMRF) was defined as the BMD. The BMD, its lower (BMDL) and upper (BMDU) bounds, and additional model fit statistics were computed for each feature. The BMD modeling module also provides interactive visualizations of models, enabling users to inspect fit quality, evaluate dose‐responsiveness, and identify genes that were frequently dose‐dependent across multiple experimental conditions. Following individual gene modeling, BMDx2 supports the computation of a transcriptome‐wide point of departure (twPOD), which summarizes the effective dose threshold across the entire transcriptome using a variety of established methods. More details on the dose‐dependent pipelines can be found in paragraph “*BMD modelling”* of the Supporting Information.

**Figure 1:**
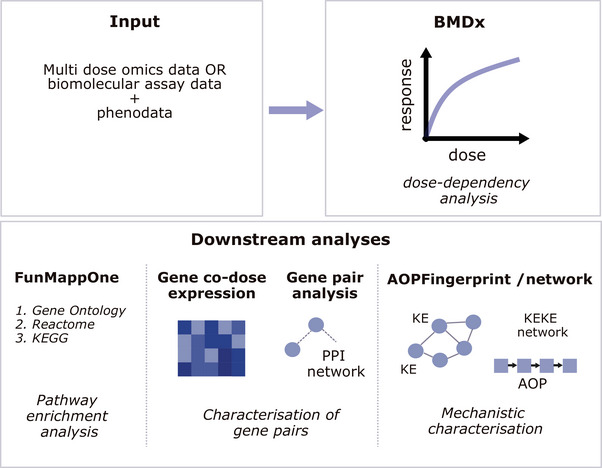
The BMDx2 tool includes four main modules: a) BMD analysis, b) comparative analysis of different experimental conditions c) AOP analysis d) co‐dose dependence analysis.

Moreover, BMDx offers downstream functionalities to characterize and further analyze the results of the dose‐dependent analysis (**Figure** **1**). In particular, pathway‐level enrichment analysis was available through the FunMappOne package.^[^ ^17^ ^]^ Co‐dose dependency analysis to identify gene pairs or gene modules exhibiting similar expression profiles across increasing doses was offered through the BMDx package. This includes pairwise correlation, clustering, and the projection of highly correlated gene pairs onto known Protein‐Protein interaction (PPI) networks, highlighting potentially co‐regulated or functionally related genes. More details can be found in the “*Gene co‐dose dependency” and “Biological interaction network”* paragraphs of the Supporting Information.

Finally, mechanistic interpretation was extended through the annotation of dose‐responsive genes with known KEs and AOPs using the AOPFingerprintR package. This step connects molecular‐level responses to structured mechanistic frameworks relevant to human health and regulatory toxicology, enabling mechanistic fingerprinting of chemical exposures. More details on data and implementation were available “*AOPFingerprintR Analysis: KE and AOP annotation and Enrichment*” of the Supporting Information and Figures S4–S7 (Supporting Information). For a detailed description of the BMDx2 implementation, the full analysis pipeline, and additional methodological considerations please refer to the Supporting Information.

### 2.2 Statistical Analysis

The transcriptomic and the differential analysis data for the multi‐walled carbon nanotubes (rCNT) case study were obtained from the original study.^[^ ^18^ ^]^ Similarly the RNA‐Seq and the in vitro data for bleomycin exposure comes from the original paper.^[^ ^19^ ^]^

VST transformation was applied to the RNA‐Seq data with the DESeq2 package. Dose‐response modelling was performed using the functions from the BMDx R package for the rCNT and in vitro datasets, while the BMDx2 app was used for the RNA‐Seq data. Poorly fitting models (*R* ^2^ < 0.6) were excluded. Optimal model selection was performed by using the AIC method for both the RNA‐Seq case study and the in vitro dataset, instead model averaging was applied to the rCNT case study. BMDx utility functions, based on Pearson correlation analysis, were used to correlate dose‐response profiles of gene expression and methylation data across 1000 uniformly spaced doses. Enrichment analyses were conducted using gprofiler2^[^ ^20^ ^]^ and Funmappone^[^ ^17^ ^]^ (Fisher's one‐tailed test FDR‐adjusted p‐value < 0.05 or Set Counts and Sizes‐adjusted p‐value threshold of < 0.05), and AOPFingeprintR packages (Fisher test, FDR adjusted p‐value < 0.05).Pearson Correlation between gene pairs was computed with the R Stat package, N = 1000. More details were provided in the Supporting Information.

## 3 Results and Discussion

BMDx2 is an R‐Shiny software with a user‐friendly interface, designed to support comprehensive, mechanistically informed, and reproducible exploration and evaluation of dose‐dependent effects in toxicogenomic datasets (Figure 1). It accommodates multiple data types, including transcriptomics, DNA methylation and targeted in vitro test data. The platform requires data where multiple doses have been tested and supports the joint analysis of multiple experimental conditions. BMDx2 uniquely integrates benchmark‐dose modeling with AOP mapping, extending beyond pathway enrichment to deliver regulatory‐relevant insights (Table S2, Supporting Information). Compared with existing tools for BMD analysis of omics data (Table S2, Supporting Information), BMDx2 introduces a series of methodological and functional innovations that provide mechanistic interpretation of chemicals effect through the AOP framework. Among other tools, BMDExpress has been the most widely adopted for deriving transcriptome‐wide points of departure and has been applied in several studies to support mechanistic and AOP‐related analyses.^[^ ^21^, ^22^, ^23^ ^]^ However, despite its robustness for dose–response modeling, BMDExpress does not natively implement gene‐to–key event (KE) mapping, AOP enrichment analysis, or network‐based visualization of mechanistic relationships. In contrast, BMDx2 provides functionalities for both dose–response analysis and KE/AOP enrichment analysis through the BMDx and AOPFingerprint modules, which are also available as an R package and via APIs. This dual implementation enables users to choose between a standalone graphical user interface (GUI) for ease of use, or modular integration into customized workflows, thereby facilitating interoperability and adaptability to specific analytical needs. Finally, when compared with other available tools, BMDx2 is the only platform that enables pairwise gene comparisons, advancing toward a systems biology perspective in which gene–gene interactions can be explored to interpret the complex biological responses elicited by chemical exposures.

In the following sections, we showcase the versatility and practical utility of BMDx2 through case studies, which feature well‐studied, data‐rich chemicals tested at a minimum of three doses. The configuration of the case studies was designed to demonstrate the flexibility of BMDx2 in offering parameter settings that can be tailored to diverse analytical needs. See section “*Case studies*” in Supporting Information for more details. The following paragraphs describe two case studies, one using multi‐omics data (DNA microarray and DNA methylation) and another using RNASeq data. A third case study using cytokine expression and qPCR data is reported in section *“Case study Targeted In Vitro Data”* of the Supporting Information.

### 3.1 Multi Omics Case Study

Understanding the biological responses to chemical exposures by integrating transcriptomic and DNA methylation data provides a powerful mechanistic view of the chemical MoA. While multiple studies exist exploring the response to nanomaterials, covering multiple omics dimensions,^[^ ^24^, ^25^ ^]^ there is a lack of methods that can fully exploit omics integration to uncover for example how transcriptional dynamics and epigenetic regulation evolve together into coordinated toxicological responses. In this case study, we showcase how the BMDx functionalities can be used to perform such integration in the context of dose‐dependent analysis. Using rigid multi‐walled carbon nanotubes (rCNTs), a well‐studied and data‐rich nanomaterial with a known MoA, we demonstrate how BMDx enables the mechanistic interpretation of multi‐omics responses, identifying functional and temporal patterns that are directly relevant to toxicological assessment.

#### 3.1.1 Multi‐Omics Sensitivity Analysis of Dose‐Dependent Genes Links the Transcription and Methylation Mechanisms of Action of rCNTs

In this case study, we analyzed datasets including transcriptomic and DNA methylation data from THP‐1 macrophage cell lines exposed to rCNTs at 5, 10, and 20 µg mL^−1^ over 24, 48, and 72 h.^[^ ^18^ ^]^ More details can be found in section “*Multi‐omics rCNT data collection*” of the Supporting Information. Dose‐dependent analysis was performed separately for each data type (section “*BMDx analysis of rCNT data”* of the Supporting Information). At the transcriptomic level, 4023, 4474, and 4157 dose‐dependent genes (DDGs) were identified at 24, 48, and 72 h, respectively (Table S3, Supporting Information). For DNA methylation, 185, 219, and 227 dose‐dependent promoter regions were found at the same respective time points (Table S4, Supporting Information). Across time, 2870 genes were persistently altered dose‐dependently, while only four promoter regions showed persistent dose‐dependent DNA methylation variations (Figure S8, Supporting Information).

Sensitivity, defined by lower BMD values, was compared across omics layers and time points. At 48 h, both gene expression and DNA methylation data showed the lowest BMDs compared to the 24‐ and 72‐h time points (Figure S9, Supporting Information). The functional roles of genes in the two omics layers indicate a shift from acute inflammatory regulation to gene expression regulation, suggesting that the lower BMD at 48 h might reflect a convergence between regulatory priming and downstream effects in THP‐1 cells upon rCNTs treatment (Tables S5–S7, Supporting Information). In the transcriptomic data, sensitivity increased over time with a lower BMD at 72 h than at 24 h. Conversely, DNA methylation showed higher sensitivity at 24 h than at 72 h (Figure S9C,D, Supporting Information), highlighting distinct temporal dynamics between the two omics layers.

We hypothesized that differences in dose sensitivity between transcriptional and DNA methylation responses might reflect distinct layers of cellular regulation, from rapid adaptations to longer‐term changes. We also explored whether integrating these layers could improve understanding of the rCNT MoA over time.

**Figure 2:**
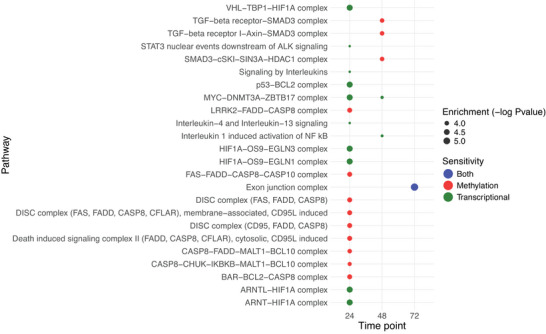
Temporal and mechanistic partitioning of dose‐sensitive gene regulation following rigid multi‐walled carbon nanotubes (rCNTs) exposure. (A) Pathway enrichment bubble plot showing dose‐dependent sensitivity of genes with the lowest estimated benchmark dose (BMD) at the transcriptomic and methylation levels across 24‐, 48‐, and 72‐h post‐exposure to rCNTs. Number of dose dependent genes at the transcriptomic level are 4023 at 24 h, 4474 at 48 h and 4157 at 72 h; number dose dependent genes at the methylation level are 185 at 24 h, 219 at 48 h and 227 at 72 h; enriched terms were identified with Fisher's one‐tailed test and the significancy is based on FDR‐adjusted p‐value threshold of < 0.05.

To test this hypothesis, we identified DDGs in common between transcriptomics and DNA methylation at each time point and classified them by greater sensitivity in either layer. A total of 111, 148, and 147 such genes were found at 24, 48, and 72 h, respectively (Figure S10, Supporting Information). Sensitive genes at the transcriptional level at 24 h and 48 h were enriched in stress‐related pathways (*HIF1A*, *MYC*, *TP53*), inflammation regulators (*IL6*, *STAT3*) and programmed cell death (*CASP8*) (**Figure** **2**; Table S7, Supporting Information). This suggests a fast, transient response regulated mainly by early transcription factors, such as *TP53*, which is activated independently of methylation.^[^ ^26^ ^]^ Many enriched functions, such as the MYC‐DNMT3A‐ZBTB17 complex, are part of rapid‐response hubs linking transcriptional activation to epigenetic reprogramming (Table S7, Supporting Information).^[^ ^27^ ^]^

Conversely, genes most sensitive at the DNA methylation level were enriched in functions related to cell death, immunological response, as well as calcium signalling, which is closely tied to long‐term transcriptional regulation and epigenetic plasticity (Table S7, Supporting Information).^[^ ^28^ ^]^ Interestingly, *TGF‐beta/SMAD3* and *SMAD3/HDAC1* complex pathways were enriched by genes more sensitive to DNA methylation at 48 h (Figure 2; Table S7, Supporting Information). The SMAD3 complex is essential in the *TGF‐beta* canonical pathway and its activation during priming and differentiation starts widespread genomic methylation.^[^ ^29^ ^]^ As DNA methylation is a more stable epigenetic mark than transient transcription factor binding,^[^ ^30^ ^]^ the methylation sensitivity observed at 48 h in a pathway linked to chromatin remodelling and transcriptional repression, suggests a mechanism for locking in specific gene expression states. Such stability could help sustain transcript expression over time, with methylation acting act as a long‐term stabilizer of transcriptional programs, particularly with rCNT exposure where chronic inflammation demands gene expression changes on a longer time frame. Notably, *TGF‐beta* is known to have a key role in the progression of lung fibrosis, a well‐known effect of chronic exposure to rCNT.^[^ ^18^, ^31^ ^]^ Consistent with our findings, the TGF‐beta/SMAD3 complex cascade has recently been described as tightly linked to epigenetic rewiring of macrophages inflammatory differentiation as well as in fibroblasts, reinforcing and stabilising transcriptional programs in lung fibrosis.^[^ ^32^, ^33^ ^]^ Notably, this epigenetic regulatory axis has been confirmed as the critical driver of fibroblast‐myofibroblast transformation and, consequently, of fibrosis progression.^[^ ^34^ ^]^

The Exon Junction Complex was the only function found to be sensitive to both methylation and transcription at 72 h (Figure 2; Table S7, Supporting Information). This overlap may reflect a role for RNA processing and export in the consolidation of long‐term gene expression patterns.^[^ ^35^ ^]^ These findings highlight the potential of the BMDx2 tool to discern mechanistic differences between omics layers sensitivity, offering valuable insights into the temporal and functional dynamics underlying dose‐dependent cellular responses to rCNT.

#### 3.1.2 Multi‐Omics Dose‐Dependent Analysis Reveals Temporally Distinct Regulatory Layers of Individual Gene *Loci*

**Figure 3:**
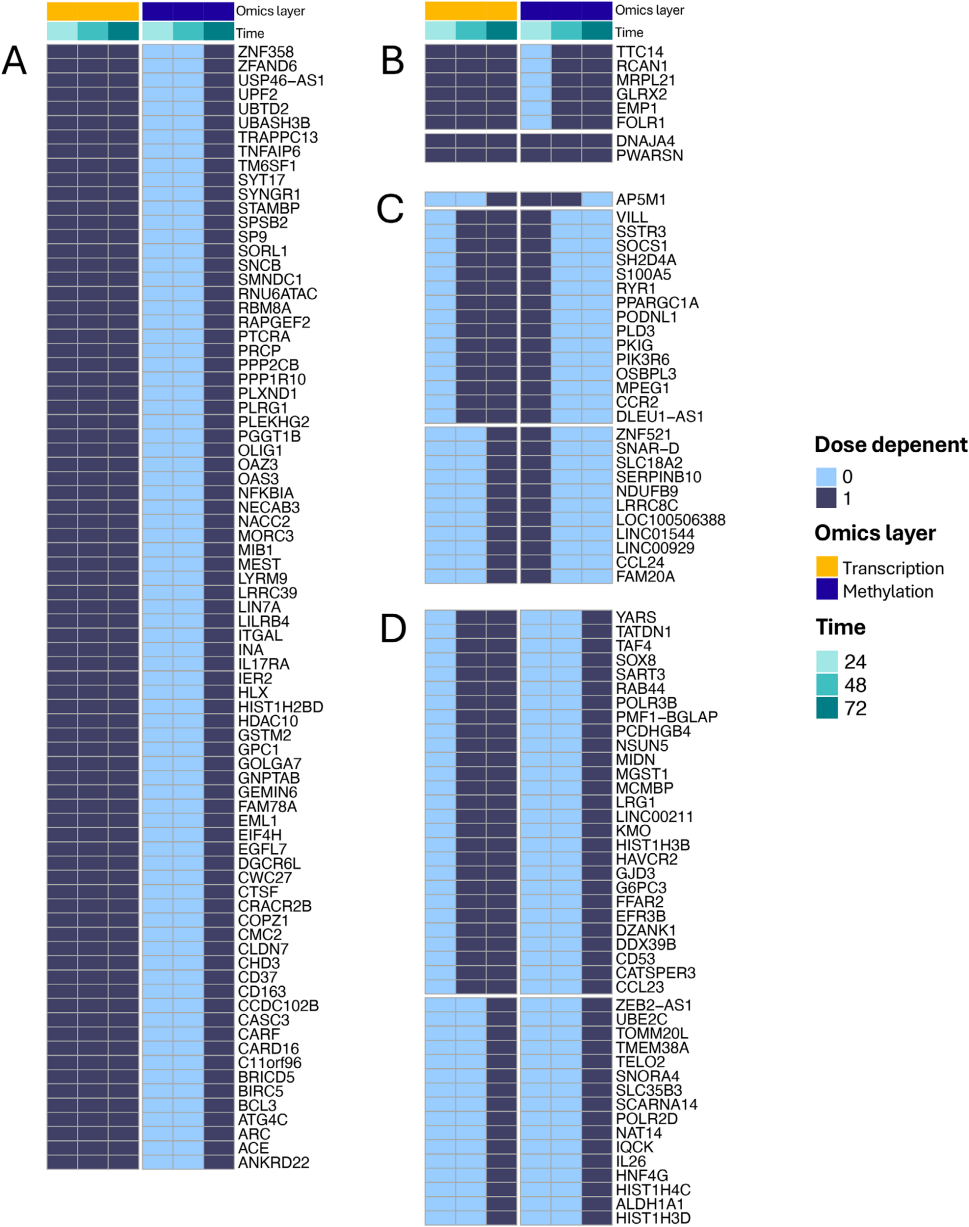
DDGs with various sensitivity patterns across time points and omics layers. A: dose‐dependent sustained in transcription and late in methylation. B: overall sustained transcription and methylation. C: dose‐dependent late in transcription and early in methylation D: dose‐dependent late in transcription and late in methylation.

By integrating multi‐omics sensitivity analyses with the BMDx package functionalities, we uncovered temporal patterns indicating that epigenetic shifts can mark the transition from transient to more stable gene regulation, either sustaining prolonged expression or enforcing silencing once an acute response is no longer needed. Therefore, we further hypothesized that genes showing sustained, dose‐dependent deregulation may undergo promoter methylation changes detectable at later time points, particularly at 72 h. Genes with such patterns across omics layers could be especially relevant for understanding long‐term biological alterations. Thus, we systematically analyzed all combinations of gene expression and promoter methylation dose‐dependency patterns across 24, 48, and 72 h (**Figure** **3**). We identified distinct classes of genes based on their temporal response profiles, to characterize time‐ and activity‐dependent epigenetic transitions and their potential mechanistic implications (See section “*BMDx analysis of rCNT data*” of the Supporting Information for more details).

Functionally, many of the transcriptionally sustained genes (i.e., genes that are always dose‐dependent at all studied time points in transcriptomics) were implicated in core stress response pathways and are consistent with the established MoA of rCNTs in macrophages (Figure 3; Figure S11, Supporting Information).

Genes such as *IL17RA*, *NFKBIA*, and *ITGAL*, involved in proinflammatory signalling and cell migration, displayed sustained transcriptional induction. Chronic upregulation of these genes may contribute to prolonged immune activation and tissue inflammation.^[^ ^36^, ^37^ ^]^ The subsequent promoter methylation observed at later time points may reflect an epigenetic feedback mechanism aimed at attenuating this inflammatory drive and limiting potential tissue damage. rCNT exposure in macrophages is known to trigger frustrated phagocytosis and lysosomal destabilization, leading to oxidative stress and pro‐inflammatory cytokine production.^[^ ^7^, ^38^ ^]^ These functions are evident as key events in which the identified genes are mapped (Figure 3A; Figure S11, Supporting Information). The later‐stage methylation of inflammatory genes may represent an adaptive shift from an acute immune response toward resolution or immune tolerance. Genes involved in ECM and tissue remodelling, such as *TNFAIP6*, *ACE*, and *SORL1*, also showed this dynamic multi‐omics pattern. While beneficial during tissue repair, their persistent activation could drive pathological tissue remodelling (e.g., scarring) or sustain the pro‐inflammatory microenvironment underlying fibrosis.^[^ ^18^ ^]^ Promoter methylation may act here to modulate or terminate the repair program, particularly under sustained exposure.

Persistent responses to oxidative and metabolic stress may necessitate transcriptional attenuation, potentially achieved through promoter methylation at 72 or already at 48 h (Figure 3A,B). This is exemplified by genes such as *HDAC10* and *UBTD2*, involved in chromatin remodelling and proteostasis. Indeed, functional annotation of genes with sustained transcription and later methylation patterns and is further supported by enrichment analyses showing sustained oxidative stress responses, that is epigenetically regulated at 48 and 72 h^[^ ^39^, ^40^ ^]^ (Figure S11, Supporting Information). The antiviral response gene *OAS3*, an interferon‐stimulated gene, also displayed dose‐dependent activation. While protective during acute stress, chronic induction may be detrimental, with methylation potentially serving to prevent overactivation of innate immune pathways at later time points.^[^ ^41^ ^]^ Similarly, the consistent induction of key components of the nonsense‐mediated decay and mRNA surveillance machinery (*UPF2*, *RBM8A*, and *CASC3)*, points to prolonged pressure on translational fidelity, reflecting proteotoxic burden or transcriptional instability, which the cell could counterbalance through epigenetic repression.^[^ ^42^ ^]^

It is interesting to note that some genes, including *SOCS1* and *PPARGC1A*, show an early methylation alteration followed by a sustained gene expression alteration (Figure 3C). The role of these genes as metabolic switch and their related functions (Figure S11, Supporting Information) may suggest a dynamic regulation in which epigenetic mechanisms may initiate metabolic reprogramming under dose‐dependent stress conditions.^[^ ^43^, ^44^ ^]^ In macrophages, such epigenetically driven metabolic shifts are known to promote the persistence of a pro‐inflammatory state and contribute to fibrosis progression by supporting fibroblast activation and ECM deposition.^[^ ^45^ ^]^

Finally, we identified a subset of genes exhibiting late (48–72 h) regulation in both expression and promoter methylation, indicating their potential roles in long‐term cellular adaptation or fibrotic transformation (Figure 3D). Of particular interest is *SOX8*, a member of the SOX family of transcription factors which have previously been implicated in fibrosis in both the heart and lung.^[^ ^46^, ^47^ ^]^

Collectively, these results show how rCNT exposure induces an intertwined dynamic molecular response in macrophages. An early transcriptional burst enables rapid adaptation to oxidative, proteostatic, and inflammatory stress. Persistent activation of these pathways may herald the transition to cellular dysfunction, including cytotoxicity, membrane disruption, and apoptosis, as evident from the mapped functionalities and associated key event annotation (Figure S11, Supporting Information). The delayed onset of promoter methylation appears to serve as a mechanism of epigenetic adaptation, potentially supporting long‐term reprogramming of macrophage function in the context of sustained exposure, or potential recovery.^[^ ^32^ ^]^ This layered response underscores the added value of integrating multi‐omics data to capture temporal dynamics and mechanistic transitions from acute stress responses to epigenetically encoded outcomes.

Building on the observed temporal link between transcription and methylation, we used BMDx correlation functionalities to test whether this association also holds for individual loci, reflecting a biologically meaningful coupling between transcriptional activity and later epigenetic regulation in the same gene (See section “*BMDx analysis of rCNT data*” for more details). More specifically, we used the functionalities of BMDx to correlate the gene expression and methylation status of altered genes at 24, and 72 h, respectively (Figure S12, Supporting Information).

As expected, two distinct regulatory patterns emerged. At 72 h, most genes (including the previously discussed *NFKBIA*, *IL17RA*, *CTSF*, *ZNF358*, as well as *FOXO6*, and *HIST1H2BD*) exhibited an inverse correlation between expression and methylation. This behaviour is expected and represents the canonical relationship in which sustained transcriptional activity is ultimately modulated (i.e., either dampened or reinforced) through promoter methylation.^[^ ^48^ ^]^ The functional role of these genes in hyperinflammation and reprogramming is well aligned with the chronic effects of MWCNT, which can ultimately lead to permanent tissue scarring and fibrosis.^[^ ^18^, ^49^, ^50^ ^]^ These findings give a molecular and mechanistic perspective on the long‐term response, as well as the system recovery that may serve to mitigate potential damage from persistent activation of stress pathways and to restore cellular homeostasis.

In contrast, a smaller but functionally relevant subset of genes, including *FAM69*, *CASC3*, *FOLR1*, and *PPP2CB*, showed a concordant directionality in both expression and promoter methylation. These non‐canonical patterns suggest that methylation in these genes may be involved in different types of transcriptional regulation. For instance, *CASC3*, a core component of the exon junction complex, may require sustained activity of the mRNA surveillance machinery under conditions of proteotoxic stress.^[^ ^51^ ^]^ Similarly, *PPP2CB*, involved in signal transduction and cell cycle control, may be persistently activated to buffer stress responses, with methylation marking transcriptionally active regions.^[^ ^52^ ^]^ While this relationship does not follow the common assumption of increased methylation leading to decreased gene expression activity, it is now well known that methylation can have various effects on gene transcription, often associated with the location of the methylation on the genes.^[^ ^53^ ^]^

In conclusion, in this case study we have exploited the novel BMDx functionalities to study the mechanism of response to rCNTs from biological processes to the individual loci level, across two different omics layers. This case study has showcased how a more advanced use of the BMDx tool can uncover the complex and temporally dynamic relationship between gene expression and DNA methylation in macrophages responding to rCNTs. It illustrates how integrating multi‐omics data over time can reveal complex molecular responses to nanomaterials that point to THP‐1 cellular reprogramming leading to long‐term fibrosis.

### 3.2 Transcriptomics Case Study

In the previous case study, we demonstrated how analysing multi‐omics data with our novel framework can unravel the MoA of rCNTs and link it to lung fibrosis integrating both transcription and DNA methylation. This multiscale approach provides unprecedented mechanistic insights, especially valuable in research settings. It does, however, rely on expert‐driven interpretation and prior biological knowledge, making it less straightforward to apply in standardized contexts.

In the present case study, we instead apply a more standardized and less user‐dependent analysis, aligned with regulatory needs. Building on the results of the previous case study indicating fibrosis as an outcome, here we showcase how using the systems‐biology based functionalities of BMDx2, we can reconstruct the profibrotic potential of a well‐characterised profibrotic chemical, bleomycin, starting from the transcriptomics data layer alone, from the same THP‐1 cell line biological system.^[^ ^54^, ^55^, ^56^ ^]^ Specifically, we use gene co‐dose dependency analysis and mapping of molecular alterations to the AOP framework to demonstrate how BMDx2 can support the interpretation of fibrosis‐related mechanisms in a reproducible and regulatory‐friendly manner.

We used publicly available transcriptomics data,^[^ ^19^ ^]^ generated through RNA‐Seq analysis of THP1 cells exposed to bleomycin at six concentrations (0, 20, 40, 60, 80, 100 µg mL^−1^) over three time points (24, 48, and 72 h), which were pre‐processed with variance‐stabilizing transformation (VST) prior to downstream analysis (Figure S13, Supporting Information). To quantify the ddMoA of bleomycin, we investigated the number of genes that show a dose‐dependent alteration at the three time points. The results show a dynamic shift in the number of DDGs across the time course, with fewer DDGs at 24 h (154 genes), a marked increase at 48 h (422 genes), and a plateau at 72 h (401 genes). The list of DDGs, and their estimated BMD, BMDL and BMDU values can be found in Table S8 (Supporting Information). MoA characterization was performed through enrichment analysis of the DDGs in biological pathways using the FunMappOne module (Figure S14, Supporting Information), together with a gene set enrichment analysis based on genes ranked by their frequency (Figure S15 and Table S9, Supporting Information). Details on how BMD modelling is performed for this case study can be found in section “*BMDx2 analysis of bleomycin data*” of the Supporting Information.

#### 3.2.1 Co‐Regulated Dose‐Dependent Modules Highlight Bleomycin Induced Chronic Inflammation

To better understand coordinated cellular responses, it is essential to move beyond individual gene analysis and examine how sets of coordinated genes contribute to the response to bleomycin. We hypothesized that genes exhibiting similar dose‐response profiles are co‐regulated and participate in common biological functions that can explain different components of the bleomycin MoA. We used the gene pair functionality of the BMDx2 tool to compare and cluster the correlation profiles of all genes at the transcriptomic level at 72 h. We also performed enrichment analysis of the resulting clusters to characterize the biological processes in which the genes are involved, within the same BMDx2 module. As a result, we discovered that the 401 DDGs at 72 h grouped into three clusters (Table S10, Supporting Information). These clusters of co‐regulated genes reflect the activation of specific cellular programs in response to prolonged bleomycin exposure (Figure S16, Supporting Information). Cluster 2 enriches events indicative of a shift toward cell cycle arrest and DNA repair, consistent with the primary genotoxic effect of bleomycin. Conversely, Cluster 1 reflects the upregulation of signaling pathways that support cellular stress response, particularly inflammatory processes triggered by chemical exposure and DNA damage. Prolonged and sustained activation of these inflammatory responses, over time, may contribute significantly to the development of fibrosis, similar to the mechanism described for rCNTs.^[^ ^57^ ^]^ Details on gene co‐expression analysis, clustering and functional annotation performed in BMDx2 can be found in section “*Gene co‐dose dependency*” of the Supporting Information and in the BMDx2 manual.

**Figure 4:**
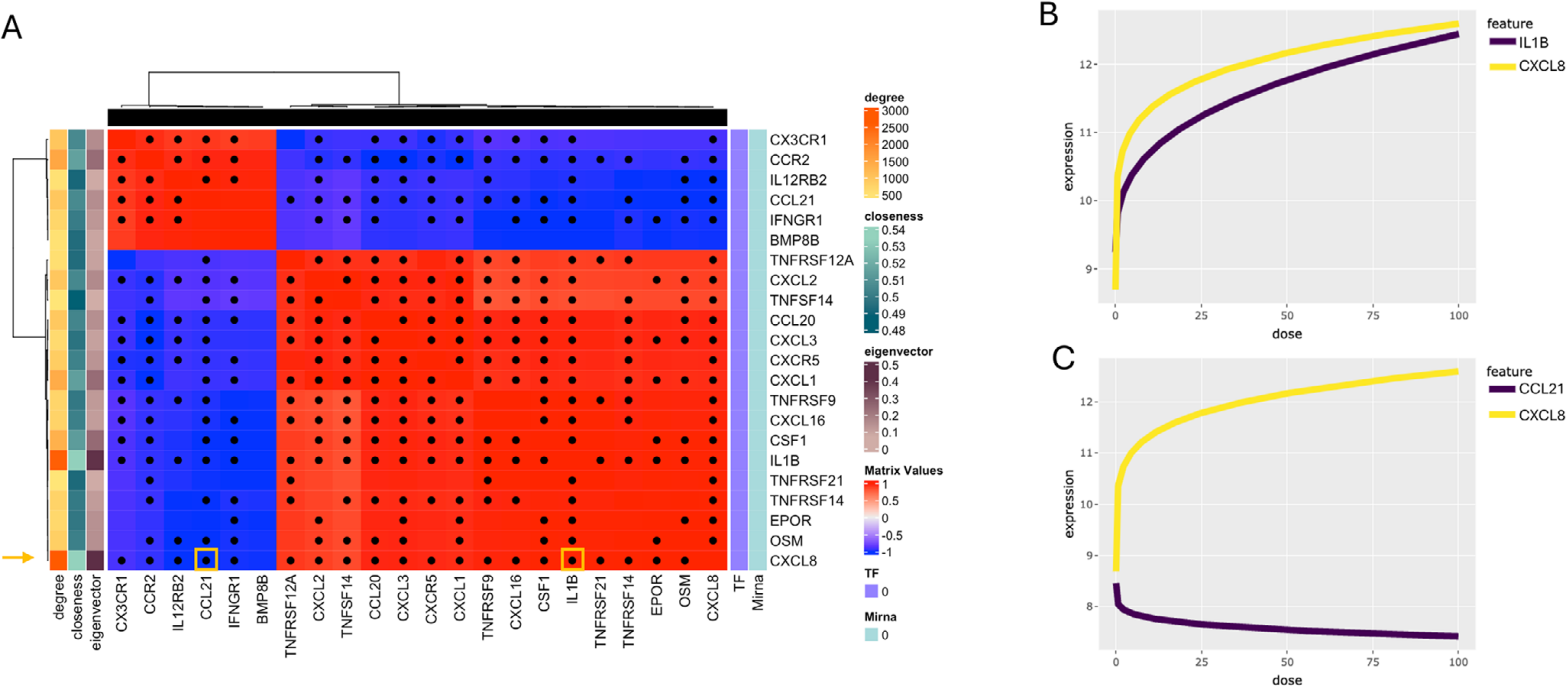
A) Gene pair analysis of the genes mapped in the Cytokine‐cytokine receptor interaction KEGG pathway (at 72 h). The color gradient (from blue to red) indicates the value and direction of Pearson correlation between gene pairs, while black dots mark known PPI interactions. Transcription factor (TF) status and PPI network features (degree, closeness, eigenvector centrality) are also labeled. CXCL8 is highlighted with an orange arrow, and its interactions with CCL21 and IL1B are marked by orange rectangles. B,C) show the predicted expression response curves for CXCL8 in combination with IL1B and CCL21, respectively, across varying doses. Curves in panel B and C show 0.98 and ‐0.99 Pearson correlation with pvalue<1e‐5 after two tailed t‐test, sample size N = 1000.

The development of complex phenotypes, such as fibrosis, arises from the interplay of multiple lung cell types and depends on intercellular communication.^[^ ^58^ ^]^ We propose that profibrotic chemicals engage cytokine–receptor signaling, generating co–dose‐dependent gene expression patterns, with cytokines acting as central mediators of phenotype development. Thus, we analyzed the gene‐gene interaction of all the DDGs from the enriched Cytokine‐cytokine receptor interaction pathway at 72 h (**Figure** **4**A). BMDx2 builds the correlation matrix between expression response curves of genes belonging to the Cytokine‐cytokine receptor interaction pathway. Moreover, the correlation matrix is also enriched with additional molecular layers, including transcription factor status and protein–protein interaction (PPI) statistics (Figure 4). Information on physical connections between gene‐associated proteins is integrated into an interactive heatmap for cluster visualization, which can also be applied to predefined gene groups.

For example, CXCL8 shows high PPI centrality, connecting to many proteins, and its expression correlates both positively and negatively with genes from the two clusters (Figure 4A). A positive correlation with IL1B (Figure 4A,B) aligns with their roles as NF‐κB‐regulated pro‐inflammatory mediators: IL1B stimulates *CXCL8* expression, amplifying neutrophil recruitment and inflammation.^[^ ^59^ ^]^ Notably, the *CXCL8* axis, which is activated downstream of *IL17RA‐NFkB* signaling, a central responder to both rCNT and bleomycin, is further regulated by *SOCS1*.^[^ ^60^ ^]^ The early dose‐dependent methylation observed in response to rCNT suggests that *SOCS1* may have a critical role in controlling the amplitude and persistence of the cytokine and chemokine responses. In contrast, CXCL8 negative correlation with CCL21 (Figure 4A–C) reflects their distinct immune roles. While CXCL8 is primarily associated with acute innate inflammation, CCL21 plays a key role in adaptive immunity by directing T cell and dendritic cell migration to lymphoid tissues.^[^ ^61^ ^]^ A decrease in CCL21 expression concurrent with elevated CXCL8 levels may reflect a transition toward an innate‐dominated immune response, consistent with the early inflammatory phase of bleomycin‐induced fibrosis.^[^ ^57^, ^62^ ^]^

Both the clustering and PPI based gene pairs analysis confirmed bleomycin induces a prolonged inflammation, which can be considered the hallmark of the compound profibrotic effect. These results resemble those of the previous case study on rCNT on multi‐omics, where uncontrolled and prolonged inflammation in the transcriptome is epigenetically regulated and induces fibrosis in long‐term settings.

#### 3.2.2 Mapping of Dose‐Dependent Transcriptomic Profiles to the AOP Framework Mechanistically Explains the Profibrotic Effects of Bleomycin

Understanding the MoA of chemicals is a cornerstone of predictive toxicology and human health risk assessment. Here we hypothesized that mapping the ddMoA of bleomycin to the AOP framework would offer a comprehensive map of the biological processes and functional links that lead to lung fibrosis upon bleomycin exposure. Here, we used the AOP fingerprinting functionality to summarize the ddMoA at the level of KEs and AOPs, for each time point present in the experimental data of THP‐1 exposure to bleomycin (Figure S17, Supporting Information). This visualization highlights AOPs consistently enriched across time points, allowing the assessment of enrichment strength (p‐value), and the number of contributing events within each AOP. The enriched AOPs primarily represent tumor cytotoxicity and fibrosis/lung fibrosis, consistent with the known profibrotic activity of the drug, while also detailing its known cytotoxic impact on tumor cells. Enrichment of these AOPs at 72 h, with early cytotoxicity signals at 24 h, underscores the dynamic nature of the MoA of bleomycin^[^ ^19^, ^54^ ^]^ and provides temporal insights into the progression of its biological effects.

**Figure 5:**
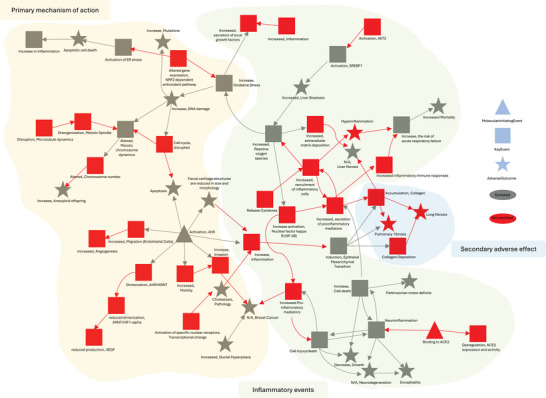
Subnetwork of Adverse Outcome Pathways (AOP) Network generated from DDGs at 72 h. MIEs are represented as triangles, KEs as squares, and AOs as stars. Enriched events (N = 401 of dose‐dependent genes at 72 h, FDR‐adjusted *p*‐value threshold of < 0.05, Test = Fisher test) are highlighted in red, while non‐enriched events are shown in gray.

While AOP fingerprints offer a snapshot of enriched AOPs, they do not capture the complex interplay between KEs that spans across multiple AOPs. In contrast, modeling the MoA as a network of interconnected KEs significantly increases the information content of the analysis, allowing simultaneous investigation of multiple toxicity mechanisms and the prediction of adverse outcomes, consistent with systems biology principles which emphasize the importance of connections over isolated elements.^[^ ^6^ ^]^ Starting from the DDGs at 72 h we reconstructed the corresponding KE‐KE network (**Figure** **5**). To get a more comprehensive view of the mechanisms in the KE‐KE network we also included KEs that are not enriched by the DDGs, but are on the paths connecting them to MIEs and AOs.^[^ ^6^ ^]^ We identified a highly interconnected region within the KE‐KE network that clearly illustrates the canonical genotoxic MoA of bleomycin (Figure 5‐ yellow area), characterized by Reactive Oxygen Species (ROS) production, cell death, and apoptosis. Notably, these cellular injury and apoptotic cell death processes are directly linked to an increase in inflammation. Such inflammatory processes are depicted in the KE‐KE network as a sustained cycle of inflammation (Figure 5 ‐ green area), driven by a progressive increase in inflammatory mediator release and inflammatory cell recruitment. This persistent inflammatory response, a detrimental effect of bleomycin, forms the molecular and cellular foundation for its potential side effects. Thus, this inflammatory cluster is directly associated with the accumulation of collagen, a KE in fibrosis development. It is well‐established that chronic inflammation can induce the accumulation of extracellular matrix (ECM) components, ultimately leading to scar formation and loss of tissue function.^[^ ^63^, ^64^ ^]^ Indeed, the KE‐KE network visually demonstrates how this sustained inflammatory state can induce fibrosis in the exposed tissues (Figure 5‐ blue area). Overall, the KE‐KE network clearly shows how inflammatory events (Figure 5‐ green area) serve as the crucial bridge connecting the primary MoA of bleomycin (Figure 5‐ yellow area) to the adverse side effects of the drug (Figure 5 ‐ blue area). This comprehensive representation underscores the significant potential of studying AOPs as intertwined events rather than isolated chains, thereby enhancing mechanistic understanding and enabling a more effective evaluation of potential adverse effects.

**Figure 6:**
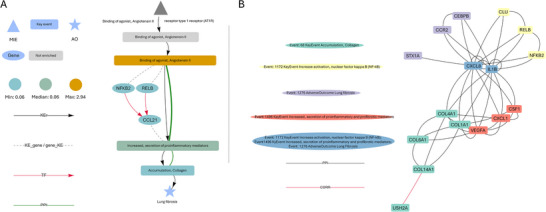
A) Enriched KEs (N = 401 of dose‐dependent genes at 72 h, FDR‐adjusted *p*‐value threshold of < 0.05, Test = Fisher test), and their associated TF, for AOP angiotensin II leading to lung fibrosis after bleomycin exposure at 72 h. KEs are depicted as nodes; non‐enriched KEs are shown in grey, while enriched KEs are color‐coded based on the average BMD values of the mapped genes; Standard KE relations are represented by black edges connecting the nodes. Green arrows represent PPI interactions between proteins in consecutive KEs, their thickness is proportional to the number of interacting proteins. Red arrows indicate relationships between TF and regulated genes. B) Aop 382: Angiotensin II type 1 receptor (AT1R) agonism leading to lung fibrosis. Top genes (and their interactions) enriched in the KEs after 72 h exposure. Genes in each KE are prioritized based on if they are TF, their degree, closeness and eigenvector centrality in the PPI, average correlation to other genes and lowest BMD. For each KE the top 5 genes are selected.

Given these results, we decided to further investigate the molecular basis behind bleomycin induced lung fibrosis. We focused our attention to the AOP *angiotensin II leading to lung fibrosis*, whose KEs bridge both the inflammatory and secondary adverse events of the bleomycin MoA (Figure 5 – green and blue areas). Interestingly, angiotensin II amplifies cytokine storm–like hyper‐inflammation while also contributing to vascular remodeling and fibrosis, indeed bridging the acute and chronic effect of bleomycin.^[^ ^65^ ^]^ We used BMDx2 functionalities to visualize this specific AOP at 72 h. We identified molecular‐level evidence that proteins encoded by genes in successive KEs physically interact and are likely involved in related biological processes, thus reinforcing the functional linkage between events (**Figure** **6**A). In addition, transcriptional regulatory mechanisms support the connection between *Increased activation of Nuclear factor kappa B* and *Increased secretion of proinflammatory mediators* KEs (Figure 6A). Particularly, NFKB2 and RELB, are two TF mapped to the former KE that regulate CCL21, a gene that is mapped to the latter KE.^[^ ^66^ ^]^ The presence of these TF‐gene connections further support the causal plausibility of the KE sequence, suggesting that NF‐κB signaling plays a critical regulatory role in driving inflammation‐related processes that may culminate in fibrosis induced by bleomycin.^[^ ^67^ ^]^ This type of visualization adds mechanistic depth to the AOP framework by overlaying a molecular layer of explanation to the casual links preset between KEs.

Supporting the identification of relevant genes involved in a specific mechanism and the way they interact is critical for chemical safety assessment and MOA characterization.^[^ ^10^ ^]^ By examining gene interactions in the PPI, it becomes possible to infer potential cooperative or regulatory roles that may not be evident from gene‐level annotations alone due to the complexity of the biological system.^[^ ^68^, ^69^ ^]^ Thus, we used BMDx2 functionality to retrieve the top 5 genes associated to KEs enriched in AOP *angiotensin II leading to lung fibrosis (*Figure 6B). The identification of specific biomarkers is essential to define a representative gene panel that captures the AOP, provides measurable in vitro readouts, and strengthens the translational and regulatory relevance of mechanistic findings.^[^ ^10^ ^]^ BMDx2 prioritizes genes within each KE using multiple network‐based and functional criteria, including TF status, topological properties in the PPI network such as degree, closeness, and eigenvector centrality, correlation with other KE genes, and sensitivity based on BMD values. This multi‐criterion ranking highlights genes that are both central and functionally relevant, providing candidate biomarkers or molecular anchors to enhance KE interpretability and support downstream application such as in vitro testing or chemical screening. Our analysis identified *IL1B* and *CXCL8* as central nodes, both are well‐established mediators of inflammation, but in this context, they also exhibit interactions with genes involved in collagen‐related processes.^[^ ^62^ ^]^ In line with our previous observation of *TGF‐beta/SMAD3* involvement in rCNT exposure, angiotensin II–driven activation of *TGF‐beta/SMAD3* pathway promotes fibroblast activation and collagen deposition, linking acute hyper‐inflammation to long‐term fibrotic remodeling and epigenetically stabilized gene expression programs.^[^ ^70^ ^]^ Interestingly, *ACE‐II* itself showed sustained dose‐dependent transcription, later locked‐in by DNA methylation at 72 h in response to rCNT (Figure 3A), which is consistent with the PPI‐informed angiotensin‐II AOP observed at 72 h in response to bleomycin. This suggests a potential molecular bridge between inflammatory responses and downstream fibrotic events, reinforcing their relevance as key regulatory players in the progression toward collagen deposition within this AOP.

By integrating dose‐dependent transcriptomic modeling with mechanistic AOP mapping, network‐based KE analysis, and biomarker prioritization, BMDx2 offers a comprehensive, systems‐level approach that allows researchers and regulators to uncover, visualize, and interpret the molecular mechanisms underlying chemical toxicity with unprecedented depth and clarity.

## 4 Regulatory Contextualization with BMDx2

Accurate chemical safety assessment requires both the mechanistic explanation of the chemical mechanism of action and the computation of PODs, which serve as the basis for deriving guidance values.^[^ ^71^ ^]^ BMDx2 computes tPOD at multiple levels such as *i)* individual gene, *ii)* gene sets and *iii)* transcriptome‐wide. Since there are currently no official regulatory guidelines for twPOD estimation, BMDx2 offer multiple methods for its computation, as described in Costa et al.^[^ ^72^ ^]^ and as summarized in section “*Transcriptome‐wide point of departure (twPOD)*” of the Supporting Information. This ensures flexibility across different research and assessment contexts. However, when the goal is to support regulatory purposes, such as tier‐1 prioritization where a conservative estimate is preferred to flag chemicals with higher hazard potential, methods like the 5‐th percentile offer a practical compromise. Such a method provides a good balance between being conservative and remaining robust to noisy gene‐level variation.^[^ ^72^ ^]^

In the multi‐omic case study, we showed how the distribution of tPOD at the individual gene level can be used to compare the sensitivity of the response across time points and multiple omics‐layers, identifying 48 h as the time point at which rCNT has the most sensitive response in both omics layers (Figure S9, Supporting Information). Similarly, such distributions can be compared for the bleomycin case study (Figure S18, Supporting Information), where steeper and Empirical Cumulative Distribution Function (ECDF) slopes at 24 and 48 h indicate higher potency of bleomycin at these time points, with a larger proportion of genes affected at lower concentrations. In contrast, the shallower slope and extended tail at 72 h suggest a decrease in the chemical potency across genes at this time point, indicating a wider range of concentrations needed to drive different endpoints. Thus, the effectiveness of bleomycin varies with time, perhaps indicating the engagement of counteracting regulatory biological mechanisms or then demonstrating the half‐life of bleomycin activity. Similar to gene wide tPOD distribution, the estimated twPOD varies over time, and also varies based on the method of calculation (Figure S19, Supporting Information). Currently, there is not clear consensus on which method to apply to compute twPOD, however it has been shown that gene‐level tPOD distributions are likely to follow a bimodal distribution.^[^ ^73^, ^74^ ^]^ We found that in instances where the BMD distribution lacks bimodality (Figure S19, Supporting Information ‐ 72 h), the Accumulation Plot Maximum Curvature method can lead to twPOD underestimation. This might occur due to the absence of a distinct bimodal pattern leading to an inaccurate determination of the first antimode, a critical parameter in the twPOD calculation with the accumulation method. Consequently, despite both the BMD distribution (Figure S18A, Supporting Information) and ECDF plot (Figure S18B, Supporting Information) indicating a lower potency of the chemical (i.e., a wider range of concentrations is needed to affect a higher number of genes) at 72 h, the accumulation method yielded a lower twPOD than at 24 h and slightly higher than at 48 h (Figure S19, Supporting Information). This finding underscores the necessity of considering the underlying BMD distribution when selecting a twPOD calculation method.

Together, these quantitative assessments of potency are complemented by a mapping of AOPs to the human health hazard classes defined in the current SSbD framework by the JRC,^[^ ^75^ ^]^ i.e., the CLP/GHS hazard classes (acute toxicity, carcinogenicity, mutagenicity, reproductive/developmental toxicity, respiratory and skin sensitization, specific target organ toxicity plus endocrine disruption). We used these to further characterize all the AOPs enriched by the DDGs in the bleomycin experiment for each time point. This categorization enabled the identification of the predominant mechanism through which the investigated chemical exerts its toxicity. The categorization of the AOPs enriched by bleomycin exposure revealed “Carcinogenicity” as the most prevalent hazard class, across all time points (Figure S20, Supporting Information). This is expected, as it informs on the established MOA of bleomycin, namely the induction of DNA damage which is flagged as carcinogenic, despite bleomycin acting as a chemotherapeutic agent in regular therapy, targeting cells that are rapidly dividing and DNA repair deficient.^[^ ^56^ ^]^ Notably, we also found a significant enrichment in the specific target organ toxicity lung (STO_tox_lung) category (Figure S20, Supporting Information) which aligns with the well‐documented pulmonary toxicity side effect of bleomycin.^[^ ^54^, ^55^ ^]^ The SSbD hazard classes annotation also enables the visualization of additional toxicity types associated with chemical exposure. Specifically, we observe toxicological categories involving the kidney, endocrine system, and reproductive functions. Although these effects are less extensively characterized than pulmonary or systemic toxicity, evidence suggests that treatment regimens combining bleomycin with etoposide and cisplatin can induce kidney, endocrine, and reproductive side effects.^[^ ^76^, ^77^ ^]^ While these outcomes are often attributed to combination therapy, it is plausible that bleomycin itself contributes, as reflected in our annotation to the SSbD hazard classes. Additionally, by examining the tPOD distribution, at the AOP level, within the SSbD hazard classes, we observe an overall reduction in chemical potency over time. This is reflected in the higher tPOD values, which may suggest the activation of counteracting regulatory mechanisms that help mitigate adverse biological outcomes.

While regulatory‐friendly approaches are essential to facilitate acceptance in safety assessment frameworks, equally important is the mechanistic characterization of toxicity, which enhances interpretability and reliability of the results. BMDx2 addresses both dimensions, representing a critical step toward the broader adoption of toxicogenomics for chemical safety assessment.

## 5 Conclusion

BMDx2 provides a mechanistically informed platform for dose‐dependent analysis of omics data. By integrating benchmark dose modelling with pathway enrichment, AOP mapping, and multi‐omics correlation, the tool provides a comprehensive framework for investigating chemical mechanisms of action. We demonstrated the BMDx2 versatility through case studies with rCNT and bleomycin in THP‐1 cells. Multi‐omic dose‐dependent characterization successfully identified an rCNT induced trajectory toward fibrosis, unravelling the complex interplay between transcription and DNA methylation and how it underlies long‐term effects. However, while mechanistically powerful and guided by the BMDx functionalities, this analysis still requires extensive biological interpretation. Overcoming this requirement, we demonstrate how a transcriptomic dose‐dependent analysis contextualized to KEs and AOPs, more directly captures the mechanistic basis leading to fibrosis, meeting the requirements of mechanistic toxicology as expected in regulatory settings and for the development of New Approach Methodologies. Together, these capabilities position BMDx2 as the first tool to embed AOPs into dose‐dependent analysis, providing regulatory‐friendly outputs such as tPODs and supporting the integration of SSbD hazard classes.

However, BMDx2 relies on the assumption of homoscedasticity, meaning that the variance of the data remains constant across all levels of the independent variables. While this assumption often holds for well‐normalized datasets, it may be violated in certain experimental conditions or data types. In such cases, variance‐stabilizing transformations can be applied to approximate this property. On the other hand, when the assumption remains unmet, the validity of the results may be less precise, and caution is warranted when interpreting the findings. Moreover, the current implementation employs human health relevant gene‐to–KE and gene‐to‐AOP mappings coming from Saarimäki et al. 2023.^[^ ^12^ ^]^ The list of KEs and AOPs present in this data collection represent a subset AOP‐wiki data retrieved in 2022. Even if AOP‐Wiki is internationally recognized as the official database for the most updated collection of AOPs and mechanistic information for AOP development, the data present in this collection is static and not automatically updated with the new evolving changes present in the AOP‐wiki resource. Literature‐mining and AI based approaches for extending such coverage are a potential solution to this problem but are not yet standardized or validated. As these efforts mature, the BMDx2 platform is well positioned to incorporate such emerging data and analytical advances, further strengthening its role as a comprehensive and adaptable tool for quantitative, AOP‐informed toxicogenomic analysis. In conclusion, BMDx2 represents an important step toward regulatory acceptance of omics‐based approaches and the broader transition to mechanism‐based toxicology.

## Conflict of Interest

The authors declare no conflict of interest.

## Supporting information



Supporting Information

Supplemental Table 3

Supplemental Table 4

Supplemental Table S5,S6

Supplemental Table 7

Supplemental Table 8

Supplemental Table 9

Supplemental Table 10

Supplemental Table 11

Supplemental Table 12

Supplemental Table 13

## Data Availability

The data that support the findings of this study are openly available in Zenodo at https://doi.org/10.5281/zenodo.17366287, reference number [1].
