# Supplementary material for: BMDx2: A Tool for Integrating Toxicogenomics‐Based Dose‐Dependency Analysis and AOP‐Based Mechanistic Insights
Source: Small Methods. 2025 Nov 12;9(12):e01728. doi: 10.1002/smtd.202501728 (PMC12716184; doi:10.1002/smtd.202501728)
Supplement: Supplementary file 1 — Supporting Information [file SMTD-9-e01728-s008.docx]

Supporting Information

BMDx2: A Tool for Integrating Toxicogenomics-Based Dose-Dependency Analysis and AOP-Based Mechanistic Insights

Angela Serra^1,2,*^, Michele Fratello^1^, Giorgia Migliaccio^1^, Emanuele Di Lieto^1^, Marcella Torres Maia^1^, Laura Aliisa Saarimäki^1,2^, Alisa Pavel^1,3^, Alexandra Schaffert^1^, Andreas Tsoumanis ^4,5,6^, Antreas Afantitis ^4,5,6^, Jack Morikka^1^, Giusy del Giudice^1,2^, Dario Greco^1,2,*^

[**Supporting Methods** 2](#_Toc211606039)

[**Implementation** 2](#_Toc211606040)

[**Data Analysis Pipeline** 3](#_Toc211606041)

[**BMD modelling** 3](#_Toc211606042)

[**BMR estimation for BMD computation** 5](#_Toc211606043)

[**Optimal model selection** 7](#_Toc211606044)

[**Akaike Information Criterion (AIC)** 7](#_Toc211606045)

[**Model average** 7](#_Toc211606046)

[**Transcriptome-wide point of departure (twPOD)** 8](#_Toc211606047)

[**The lowest dose** 8](#_Toc211606048)

[**Percentile method** 9](#_Toc211606049)

[**First mode** 9](#_Toc211606050)

[**The Accumulation Plot Maximum Curvature method** 10](#_Toc211606051)

[**Downstream analysis** 10](#_Toc211606052)

[**FunMappOne based enrichment analysis** 10](#_Toc211606053)

[**AOPFingerprintR Analysis: KE and AOP annotation and Enrichment** 11](#_Toc211606054)

[*POD Aggregation functions for KE and AOPs* 13](#_Toc211606055)

[*Visualization and molecular annotation of individual AOPs* 13](#_Toc211606056)

[*Gene Prioritization of individual AOPs* 14](#_Toc211606057)

[**Gene frequency** 14](#_Toc211606058)

[**Gene co-dose dependency** 15](#_Toc211606059)

[**Biological Interaction Network** 15](#_Toc211606060)

[**Case studies** 16](#_Toc211606061)

[**BMDx Analysis of multi-omics gene expression and DNA methylation data** **for rCNT exposure** 17](#_Toc211606062)

[**Multi-omics rCNT data collection** 17](#_Toc211606063)

[**BMDx analysis of rCTN data** 17](#_Toc211606064)

[**BMDx2 analysis of RNASeq analysis for bleomycin exposure** 19](#_Toc211606065)

[**RNASeq bleomycin data collection** 19](#_Toc211606066)

[**BMDx2 analysis of bleomycin data** 20](#_Toc211606067)

[**Results RNASeq bleomycin** 22](#_Toc211606068)

[**Characterization of the dose-response mechanism of action of chemical exposure though KEGG enrichment analysis** 22](#_Toc211606069)

[**Case study Targeted *In Vitro* Data:** 23](#_Toc211606070)

[**Case study on the Dose-Dependent Mechanisms of Action (MOA) Using Targeted *In Vitro* Data** 23](#_Toc211606071)

[**Dataset** 23](#_Toc211606072)

[**Analysis** 24](#_Toc211606073)

[**Results** 24](#_Toc211606074)

[**Dose-dependent analysis of Cytokines** 24](#_Toc211606075)

[**Dose-dependent analysis of qPCR** 25](#_Toc211606076)

[**Supplementary Figures** 27](#_Toc211606077)

[**Supplementary Tables** 43](#_Toc211606078)

[**References** 49](#_Toc211606079)

# **Supporting Methods**

## **Implementation**

BMDx2 is a comprehensive R-Shiny application for dose-dependent analysis of toxicogenomic data. It extends and integrates three core R packages (BMDx - https://github.com/fhaive/bmdx/, FunMappOne - https://github.com/fhaive/FunMappOnePackage, and AOPFingerprintR - https://github.com/fhaive/AOPfingerprintR) into a single graphical application (Figure S 1), enabling streamlined dose-response modeling, pathway enrichment, and adverse outcome pathway (AOP) mapping. While BMDx and FunMappOne were previously available only as standalone Shiny applications, their core analytical logic has now been decoupled from the graphical user interface and fully implemented as reusable R packages. In addition, AOPfingerprintR introduces an R package implementation of AOP-based mechanistic characterization of toxicogenomic data.

Designed with usability in mind, the intuitive BMDx2 GUI makes advanced analyses accessible to non-experts. It supports every stage of the analysis pipeline: from data import and preprocessing to visualization and interpretation. Intermediate results are displayed through interactive plots, and outputs can be downloaded as Excel files at multiple steps, ensuring clear and consistent documentation.

For advanced users, direct use of the underlying R packages, offers full flexibility for customization, automation, and integration of BMD modeling into high-throughput or specialized analytical pipelines. A complete description of the BMDx2 app can be found in the manual at <https://github.com/fhaive/bmdx/blob/main/manual.pdf>.

To enhance accessibility, the application also provides APIs for BMDx (<https://enaloscloud.novamechanics.com/insight/bmdx/__docs__>/), and AOPFingerprintR (https://enaloscloud.novamechanics.com/insight/aop_fingerprint/__docs__/) allowing independent hosting and integration into custom workflows or web platforms, ideal for collaborative projects or large-scale analyses. The APIs were implemented using R Plumber^[1]^, exposing endpoints that facilitate programmatic interaction with the underlying resources. To ensure reproducibility and portability across computing environments, the APIs were containerized using Docker.

## **Data Analysis Pipeline**

The BMDx2 pipeline is purpose-built for dose-response modeling in toxicogenomics, allowing researchers to investigate dose-dependent biological effects and their mechanistic underpinnings. BMDx2 offers two main functionalities: the BMD modeling analysis and the downstream analysis that allows to gain deeper biological insights based on the BMD modeling results.

### **BMD modelling**

The analysis pipeline starts with importing the experimental data and the corresponding metadata (Figure S 2A). BMDx2 is tailored for analyzing preprocessed toxicogenomic datasets, including both microarray and RNASeq data, assuming they are log2 normalized (microarrays) or variance-stabilized (VST) (RNASeq).

Afterward a pre-filtering step can be performed to identify genes demonstrating dose-dependent variation (Figure S 2B), using statistical methods such as ANOVA, trend tests, or differential analysis. The differential analysis is based on the R *limma* package^[2]^ and it is only available for gene expression data. Filtering parameters are customizable, allowing users to tailor the analysis to the characteristics of their datasets. This step focuses the subsequent modeling efforts on genes that are most likely to exhibit biologically relevant dose-response relationships, but it can also be skipped if a complete analysis is desired or if pre-filtering has already been performed externally.

Once filtering is complete, the pipeline proceeds to perform the BMD modeling (Figure S 2C). In this step, multiple dose-response models are fitted for each gene. BMDx2 implements and extends the functionalities already available in the previous version. Among the available models there are linear and polynomial models, power models, exponential models as well as the Hill model and the Michaelis-Menten model (see Table S 1) for the full list of available models). However, differently from the older version, the nonlinear model optimization implemented in BMDx2 is based on the Levenberg-Marquardt algorithm implemented in the *minpack.lm* R package.^[3]^ In addition, we introduced in BMDx2 the possibility of also fitting all the models available in the package *drc*^[4]^ within the same interface.

After the model fitting, benchmark doses are estimated. Estimating benchmark doses (BMDs) and their confidence bounds, BMDL (lower) and BMDU (upper), is central to dose-response analysis. A key step involves selecting a benchmark response (BMR), which defines a biologically significant change, typically as a percentage above the baseline (e.g., 10%). In BMDx2, BMR can be determined via three methods: relative, absolute and standard deviation (Figure S 3). In the absolute method, the BMR is a fixed response level tied to a predefined biological or regulatory threshold, regardless of baseline variability. In the relative method, the BMR is calculated as a proportion of the predicted control response, scaling the deviation based on the baseline level to reflect a percentage change. The standard deviation method introduces a Benchmark Response Factor (BMRF), linking BMR to data variability. BMDx uses a default BMRF of 1.349 (per Thomas et al.^[5]^), representing an 11% tail shift, equivalent to a 10% increase over background.

Finally, the selected BMR is projected onto the fitted dose-response model. The corresponding dose at which the model predicts the specified response shift (BMRF) is identified as the BMD. To account for uncertainty in BMD estimation, BMDx calculates lower (BMDL) and upper (BMDU) confidence bounds using the Wald method, following the approach by Gaylor et al. as recommended by the EPA.^[6]^ These bounds reflect the range in which the true BMD is expected to fall, based on the modeled variance structure. A narrow BMDL-BMDU interval indicates high confidence, while a wider interval suggests greater variability or model uncertainty. This enhances the robustness and interpretability of dose-response assessments in BMDx2.

Since multiple models are fitted for each gene, the next step is to identify the best one that represent the dose-dependent behavior of the gene with the least uncertainty in the prediction of the effective doses (Figure 2C). Thus, goodness-of-fit metrics, such as the coefficient of determination (R²), are used to evaluate how well the models represent the data. Models with an R2 below a certain threshold can be removed since they are considered not to have a relying fitting and can then be excluded from the analysis. Additional filters ensure the stability of dose estimates, and lowest as possible uncertainty, by assessing BMD/BMDL and BMDU/BMD ratios. Monotonicity checks can be applied to confirm that the dose-response relationship follows a consistent and biologically relevant trend. See the manual for a complete list of possible filtering parameters (<https://github.com/fhaive/bmdx/blob/main/manual.pdf>). Moreover, once the models have been filtered, the optimal one can be identified using the Akaike Information Criterion (AIC)^[7]^ or by computing the average model^[8]^ of those that pass the filtering criteria (Figure 2D).

Finally, the pipeline allows the user to compute a transcriptome-wide point of departure (twPOD). BMDx2 implements multiple of these methods including: the Accumulation Plot Maximum Curvature method^[9]^, the First Mode method^[10]^, the Percentile-Based methods^[11]^, the Gene Set Mean method and the Most Sensitive Gene (lowest POD). Notably, a consensus on the optimal method and the appropriate BMD parameter (BMDL or BMD) remains elusive in the literature.^[12]^

The BMDx2 is designed to include a comprehensive summary of dose-response metrics for each gene, statistical evaluations of the models, and a wide range of functionalities for the comparison of multiple experimental data (e.g. same chemical tested at different time point, or different chemicals).

#### **BMR estimation for BMD computation**

BMDx2 offers three different methods to compute the BMR, namely the absolute method, the relative method and the standard deviation method (Table S 2). The absolute method defines BMR as a fixed response shift (BMRF) with respect to the model predicted value at control doses.

$$BMR={mean}_{0} \pm BMRF$$

The relative method expresses BMR as a percentage of the predicted control response, scaling the change relative to the baseline.

$$BMR={mean}_{0} \pm(BMRF \times{mean}_{0})$$

Where ${mean}_{0}$ is the model predicted value at control doses.

The standard deviation method adjusts the BMR based on the baseline ${mean}_{0}$ and the response variability, in terms of standard deviations $(SD)$.

$$BMR={mean}_{0} \pm(BMRF \times SD)$$

In the standard deviation method, the choice of BMR method depends on assumptions about variance. With constant variance, variability is uniform across doses and estimated from all the residuals. With non constant variance, variability is not uniform across the doses and it is estimated from the residuals of the controls. However, since variance often increases with dose, more flexible modeling is required. To address this complexity, BMDx2 incorporates two additional variance modeling options: (i) a parametric variance model and (ii) a non-parametric variance estimation approach. The parametric model assumes that higher mean responses correspond to greater variability and is implemented as a log-linear function:

$$log\left( \sigma_{i}^{2} \right)=a+b\times mean_{i}$$

Where we are modeling the variance ($\sigma_{i}^{2}$) of dose group $i$ as a linear function of the mean response in logarithmic space. The logarithmic transformation ensures that the values are always positive. Moreover, the mean response and variance functions are fitted simultaneously using generalized non-linear least squares, by means of the gnls R package.^[13]^

In contrast, the non-parametric variance estimation approach calculates variance independently for each dose group $i$ based solely on the residuals of the replicates:

$$\frac{1}{n_{i}-1}\cdot\sum_{j=0}^{n_{i}} \left( y_{ij}-f\left( dose_{i} \right) \right)^{2}$$

This method avoids assumptions about the functional form of the variance relationship and instead relies on direct computation from the observed data.

#### **Optimal model selection**

##### **Akaike Information Criterion (AIC)**

The AIC is a measure used to evaluate and compare statistical models by balancing goodness of fit and model complexity, implemented in the R stats package as

$$-2 \times loglikelihood+k \times npar$$

where *npar* represents the number of parameters in the fitted model, and k=2 for the usual AIC. The model with the lowest AIC is typically selected as optimal because AIC balances model fit and complexity by penalizing the inclusion of additional parameters. This ensures the selection of models that adequately explain the data without overfitting. Derived from information theory, AIC minimizes the expected Kullback-Leibler divergence between the true data distribution and the model's estimated distribution. By prioritizing models with the lowest AIC, a trade-off that favors predictive accuracy and parsimony can be achieved making it a robust criterion for model comparison in statistical and machine learning contexts.

##### **Model average**

An alternative for selecting the optimal model is to perform model average. BMDx implements model averaging through an AIC-based combination of models fitted to the same gene’s dose-response data. This methodology accounts for model uncertainty by integrating information from multiple models while penalizing overly complex or poorly fitting ones. By incorporating multimodel inference, the approach provides more robust and reliable dose-response estimates.

For each gene, multiple dose-response models are fitted, and their goodness of fit is assessed using AIC. To determine the contribution of each model to the final prediction, Akaike weights are computed from their respective AIC values. These weights are derived using the equation:

$$w_{i}=\frac{\exp\left( -\frac{AIC_{i}-AIC_{min}}{2} \right)}{\sum\exp\left( -\frac{AIC_{i}-AIC_{min}}{2} \right)}$$

where $w_{i}$ represents the relative weight of model $i$, penalizing models with higher AIC values while favoring those with lower AIC values.

Using these Akaike weights, the predictions for BMD, BMDL, and BMDU from each model are combined into weighted averages, ensuring that models with better fit have a greater influence. This approach allows the final predictions to integrate information from multiple models while minimizing the impact of overly complex or poorly fitting ones. The model averaging process begins with fitting multiple dose-response models to the data, after which their AIC values are calculated. The relative support for each model is then quantified using Akaike weights, ensuring that models with lower AIC values contribute more substantially to the final prediction. Once the weights are determined, model predictions for BMD, BMDL, and BMDU are aggregated into a single, weighted estimate. This process effectively balances model selection uncertainty and improves the reliability of dose-response assessments. This methodology is based on the principles of multimodal inference described in Symonds and Moussalli.^[14]^

#### **Transcriptome-wide point of departure (twPOD)**

The transcriptomic point of departure (twPOD) has been proposed as a robust approach for estimating toxicity values based on transcriptomic data.^[12]^ We implemented several complementary aggregation methods to derive a single twPOD value from gene-level benchmark dose (BMDL or BMD) data. Each method offers a distinct perspective on characterizing the departure from the baseline transcriptomic response.

##### **The lowest dose**

To estimate the twPOD while mitigating the influence of potential outliers and noise associated with relying solely on the lowest benchmark dose, we implemented two alternative approaches. The first method, the lowest-ranked subset has been implemented as in ^[15,16]^: it ranks all genes based on their BMD values, enabling users to select the x-th lowest ranked gene's BMD as the twPOD; while the default is the minimum, the 25th ranked value is commonly used for enhanced robustness.^[15,16]^ The second approach utilizes the Lowest Consistent Response Dose (LCRD), following the methodology of Crizer et al.^[17]^, to pinpoint the biological feature with the most responsive and consistent change at the lowest plausible exposure level. The LCRD is identified as the lowest BMD within a rank-ordered set, where consistency is established when the ratio between all subsequent BMDs from adjacent ranks does not exceed a user-adjustable threshold, which defaults to a maximum ratio of 1.66 (a <0.25 log difference). These methods offer alternative strategies for deriving a robust and biologically relevant twPOD from transcriptomic data.

##### **Percentile method**

We also implemented a percentile-based method for twPOD determination as in Rearedon et al. ^[15]^ This approach defines the twPOD as a specific low percentile of the distribution of benchmark doses (BMDs) across all genes in the transcriptome. Common percentile choices, as cited in the literature^[15]^, include the 10th or 5th percentile of the gene BMD values. For instance, the 5-th percentile BMD signifies that 5% of the responsive genes exhibit BMDs at or below this dose level. This statistically derived twPOD aims to balance sensitivity to early transcriptional changes with stability against the influence of single-gene outliers, thus offering a more protective measure compared to relying solely on the minimum BMD. In our tool this calculation was implemented by utilizing the *quantile* function from the stats package to compute the desired percentile from the distribution of gene BMDs.

##### **First mode**

BMDx2 also implements the Probemode method for twPOD computation as described in Pagé-Larivière et al.[10] The first mode twPOD can be estimated when the distribution of BMD values shows a bi-modal (two peaks) configuration. The first mode is considered to capture the earliest and most sensitive biological perturbations, such as cell signaling, DNA damage response or pathway activation, before broader cytotoxic, stress or immune responses dominate.[18] Because these low-dose transcriptomic changes precede overt toxicity, the first mode is treated as the most conservative and biologically meaningful estimate of the dose at which sustained transcriptional disturbance could lead to adverse effects.[10,11] To identify the first mode twPOD, we used kernel density estimation on the BMD frequency distribution to detect a dose of low density that would separate the first from the second mode. Then, the first mode is obtained by finding the dose that corresponds to the highest density in the dose range between 0 and the low density point previously identified as described in Pagé-Larivière et al.[10]

##### **The Accumulation Plot Maximum Curvature method**

We also employed a data-driven method to identify the twPOD based on the point of maximum curvature, or "knee point," in the cumulative sum of gene BMDs, as proposed in Johnson et al.^[19]^ The resulting twPOD is hypothesized to reflect the dose at which broad transcriptomic perturbation accelerates, offering an unbiased estimate of a system-level tipping point. In practice, gene BMD values are sorted, and the first antimode is identified by locating the local minimum (valley) in a density estimate. An accumulation plot, representing the cumulative sum of BMDs of affected genes versus dose, is then generated on a log-dose scale.^[19]^ The dose at the inflection point of this curve, where the slope sharply increases, signifies a transition from a limited number of genes responding to a broader, more substantial transcriptional response. To objectively identify this inflection point, we utilized the Kneedle algorithm. Notably, this approach does not require a priori selection of an arbitrary percentile; instead, it leverages the inherent shape of the response distribution to define the twPOD.

### **Downstream analysis**

The final outputs of the BMDx2 pipeline include a list of dose-dependent genes for each experimental condition (e.g. chemical, time point) with a comprehensive summary of dose-response metrics for each gene. These genes can be further analyzed to provide mechanistic insights into the observed dose-response relationships. Particularly, the following downstream analysis can be performed: i) Gene frequency ii) FunMappOne based enrichment analysis; iii) KE and AOP based mapping; iv) Gene pairs analysis.

#### **FunMappOne based enrichment analysis**

FunMappOne based enrichment analysis is comprehensively described in the tool original paper.^[20]^ Briefly, FunMappOne can be used to perform pathway enrichment analysis of the genes that are deemed dose-dependent at each experimental condition. The tool will visualize the results as a heatmap with hierarchical organization of the pathways to facilitate data summarization at different levels.

#### **AOPFingerprintR Analysis: KE and AOP annotation and Enrichment**

The dose-dependent genes are mapped to key events (KEs) and adverse outcome pathways (AOPs) using the AOPFingerprintR R package. These mappings link transcriptomic changes to specific biological mechanisms and pathways, facilitating the interpretation of the data in the context of regulatory and research applications.

The annotation of the gene lists to the KEs and AOPs, relevant for human health, come from the work of Saarimäki et al.^[21]^ The dataset related to the KE relationships comes from a previously developed knowledge graph.^[22,23]^ The annotation performed in Saarimäki et al.,^[21]^ is based on data retrieved from the AOP-Wiki repository (<https://aopwiki.org>). Specifically, AOP-related information was originally downloaded in November 2020 and subsequently updated in August 2022 through the AOP-Wiki API and associated data files. The annotation of genes to KEs performed in Saarimäki et al. ^[21]^ includes human ensemble genes mapped to the KEs/AOPs. Here, the mapping is offered for *homo sapiens, mus musculus and rattus norvegicus* genes, and is available for ensemble ids, entrez gene ids and gene symbols. The conversion between humans and mouse and rat genes, and the ids mapping has been performed using the bioMart package, starting from the gene lists in Saarimäki et al.^[21]^ The initial list mapped in Saarimäki et al^[21]^ includes 969 KEs. When performing the conversion between *homo sapiens* genes to *mus musculus,* and *rattus norvegicus* 968 KEs are left with non-empty set of genes. Event:1794 (Pin-1 activation) is not present in the list of KEs annotated to rat genes, and Event:1440 (Formation of HDL-SAA) is not present in the list of KEs annotated to mouse genes. All the others are shared between the three species annotations, with proportional size preserved across the conversion (Figure S 4).

KE enrichment is performed using the Fisher’s exact test as implemented in the function *fisher.test* from R *stats* package, for the set of dose dependent genes identified for each chemical exposure present in the experimental data at each time point, against the list of KE-related genes. Enrichment p-values can be adjusted using the false discovery rate (FDR) correction or the Bonferroni correction. The same analysis is repeated against the AOP-related genes (i.e., the union of all the genes associated to all the KEs of the AOP), to identify the enriched AOPs. Following the strategies proposed by Saarimäki et al.^[22]^, an AOP is considered significantly enriched when the AOP itself and at least a certain percentage x% (or minimum of 2 KEs when the length of the AOP was less than n) of its KEs are enriched. These parameters need to be specified by the user. Default parameters are x = 33% of enriched KEs and n=6. Furthermore, BMDx2 integrates an advanced visualization framework that enables the exploration of mechanisms of action for dose-dependent effects of chemical exposure through the KE relationship network. The tool allows to visualize the enriched KEs in the network along with KEs connecting them that are not enriched, ensuring a more comprehensive assessment of the potential exposure effects. Finally, BMDx2 also allows the comparison between the network of two conditions (e.g. same chemical tested at two time points, or different chemicals tested at the same time). The comparative analysis shows the similarity between the KE relationship networks of the two conditions (i.e. the common subgraph) and the differences (i.e. the difference between the two graphs).

Finally, in the AOPFingerprintR package, AOPs were systematically mapped to Safe and Sustainable by Design (SSbD) categories for human health using a structured categorization framework. The SSbD categories were obtained from Caldeira et al. ^[24]^ and Patinha et al.^[25]^ Each AOP was evaluated to determine its biological context by examining its full name, and the most appropriate SSbD category was manually assigned.

In particular, the categories considered in this version of the annotation are acute toxicity, carcinogenicity, endocrine disruption (human health), mutagenesis, reproductive/developmental toxicity, respiratory sensitization, skin sensitization, specific target organ toxicity. All the AOPs that were not assigned to any of these categories are marked as “uncategorized”.

AOPs describing carcinogenesis, identified by terms such as cancer, tumor, adenoma, carcinoma, leukemia, sarcoma, or mesothelioma, were categorized under carcinogenicity regardless of the target organ. Those associated with effects on the offspring or the fetus, congenital malformations, developmental processes, or adult fertility and reproduction were classified as reproductive/developmental toxicity. AOPs explicitly mentioning respiratory or skin sensitization were categorized into their respective sensitization groups. Mutagenesis was designated as the category for AOPs where mutations were the primary endpoint. For AOPs describing non-cancerous toxicity unrelated to endocrine disruption or reproductive toxicity, the specific target organ toxicity category was applied, encompassing endpoints such as immunotoxicity, neurotoxicity, cardiovascular toxicity, and toxic effects on organs like the liver, lungs, kidneys, and gastrointestinal tract, while excluding those linked to other categories. AOPs that primarily addressed endocrine-related endpoints, such as thyroid disruption or adipogenesis, but did not explicitly involve fertility or reproductive success, were assigned to endocrine disruption. Finally, AOPs describing acute mortality or cell death without further detail on downstream events were classified under acute toxicity. Some AOPs were mapped to multiple categories. Figure S 5 shows the distribution of number of AOPs for each category or combination of two categories. Furthermore, the AOPs were also mapped to the endpoints and related organ (Figure S 6, Figure S 7). This approach ensures that AOPs in the AOPFingerprintR package are effectively aligned with SSbD human health indicators, providing a framework for categorizing toxicological outcomes.

##### *POD Aggregation functions for KE and AOPs*

BMDx2 allows the aggregation of individual genes PODs to the level of KEs and AOPs. BMDx2 offers multiple functions to aggregate the BMD, BMDL and BMDU values at KEs and AOPs levels, such as the mean, median, minimum, and 5th quantile of the gene-specific BMD distribution. These options allow users to select the most appropriate aggregation method for their data and biological questions, enhancing the flexibility and robustness of the analysis.

##### *Visualization and molecular annotation of individual AOPs*

The BMDx2 tool provides an interactive framework for the visualization and annotation of enriched AOPs selected by the user. For each user-selected experimental condition and AOP, the underlying network structure is assembled, including KEs and their relationships (Key Event Relationships, KERs), utilizing curated datasets from AOP-Wiki (downloaded in April 2025). Each KE node within the network is annotated with key biological and statistical information, such as BMD, BMDL and BMDU, and adjusted p-values, as derived from gene set enrichment and dose–response modeling. To further enrich the functional context, the network incorporates supporting evidence from transcription factor (TF) and microRNA (miRNA) regulation, as well as protein–protein interaction (PPI) data, by identifying regulatory connections among genes that drive KE enrichment (The data is described in paragraph “*Biological Interaction Network”*). The visual representation uses node color to encode BMD values and employs distinct shapes to differentiate molecular initiating events (MIEs), KEs, and adverse outcomes (AOs). Edge color and width are used to indicate the type and strength of mechanistic and molecular relationships, such as direct KE connections, regulatory interactions, and PPIs. All annotations and network features are updated dynamically according to the user’s selected experiment and time point, ensuring a context-specific and comprehensive display of AOP activation.

##### *Gene Prioritization of individual AOPs*

Within each selected AOP, BMDx2 prioritizes genes associated with individual KEs using a network-based multi-criteria ranking approach. For every KE, the set of genes identified as drivers of enrichment is mapped onto the context-specific PPI subnetwork (The data is described in paragraph “*Biological Interaction Network”*). Multiple topological features are computed for each gene within this subnetwork, including degree centrality (reflecting the number of direct connections), closeness centrality (measuring the average shortest path to all other genes, indicative of information transfer efficiency), and eigenvector centrality (representing the influence of a gene based on the connectivity of its neighbors). To derive a consensus ranking, BMDx2 employs the Borda method^[26]^: each gene is independently ranked according to each centrality metric, and then the aggregate Borda score for each gene is calculated as the sum of its individual ranks across all metrics. Genes are prioritized based on their Borda scores, with lower scores signifying higher overall network importance. This approach allows for a balanced integration of different aspects of network centrality, capturing both local connectivity and broader network influence. The number of prioritized genes per KE is set by the user (with a default value of 5). Importantly, TFs are always included in the prioritized gene set, and the remaining positions are filled by the top-ranked non-TF genes according to their Borda scores. This strategy ensures that key regulatory elements are retained for downstream analysis.

#### **Gene frequency**

The analysis evaluates the relationship between gene frequency across experimental conditions and centrality within a biological interaction network. Gene frequency is determined by computing the occurrence of each gene across multiple experiments (example different time points in the same experimental dataset), constructing a binary matrix that represents gene presence, and ranking genes based on their frequency. Genes exceeding a predefined threshold (set by the user) are identified and visualized using lollipop plots (See Manual at <https://github.com/fhaive/bmdx/blob/main/manual.pdf>). This analysis assumes that genes displaying dose-dependent expression across multiple experimental conditions reflect consistent and potentially regulatory responses to perturbations. Their recurrence across datasets suggests a level of robustness and functional importance that merits further investigation. To further explore functional significance, gene set enrichment analysis (GSEA) is performed on the high-frequency genes, through the gprofiler2 package^[27]^, evaluating their association with known biological pathways.

#### **Gene co-dose dependency**

We propose a pairwise gene similarity approach to analyze gene co-expression patterns across different dose levels. The method begins by generating a user-defined number of dose values (default is 1000), spanning the minimum to maximum observed doses, to establish a uniform basis for dose-response analysis. Gene expression values are then predicted for each gene at these predefined dose levels, enabling a high-resolution assessment of dose-dependent expression dynamics. Pairwise gene similarity is quantified using two complementary metrics: Pearson correlation and Euclidean distance. Users can choose to cluster genes based on Pearson correlation alone, Euclidean distance alone, or an integrated metric combining both. To compute the combined similarity, Pearson correlation is first transformed into a dissimilarity measure by subtracting it from one, after which both similarity matrices are normalized to a unit range and averaged. Finally, hierarchical clustering is performed with the R function “*hclust”* with a default parameters and number of clusters *k* selected by the user.

Finally, on top of the hierarchical clustering displayed as a heatmap, pairs of genes that are also connected in the PPI (The data is described in paragraph “*Biological Interaction Network”*) are marked. The heatmap is also annotated for the centrality of such genes in the PPI, where centrality is quantified using degree centrality, which measures the number of direct connections, and betweenness centrality, which captures a gene’s role in connecting other nodes within the network.^[28,29]^

#### **Biological Interaction Network**

We retrieved the PPI and regulatory networks of *homo sapiens, mus musculus and rattus norvegicus* from a previously developed knowledge graph.^[23]^ These networks include associations between nodes that have been mapped to Ensembl Gene IDs and were subsequently processed to provide equivalent mappings for Entrez Gene IDs and gene symbols. The mapping was performed using BioMart annotation via the BioMart package^[30]^, enabling consistent cross-referencing across gene identifiers. For each organism, three undirected graphs were generated with nodes labeled using Ensembl IDs, Entrez Gene IDs, or gene symbols. The interaction networks integrate both PPIs and regulatory elements and were constructed using the igraph package^[31]^to support structured downstream analyses. PPI data sources include STRING, HitPredict, HuRI, HIPPIE, MINT, KEGG, PINA, Lit-BM, Reactome, SignaLink, Pharos, InnateDB, Yang-16, PhosphoNetworks, HI-union, and HI-II-14. The regulatory layer comprises interactions derived from TRRUST, TransmiR, JASPAR, miRTarBase, and TargetScan, and includes diverse molecular entities such as transcription factors, microRNAs, long non-coding RNAs (lncRNAs), pseudogenes, and protein-coding genes. This integrated resource captures a comprehensive view of molecular regulation and interaction, facilitating network-based analysis of gene activity and regulatory mechanisms across species.

# **Case studies**

In this study, we present the capabilities of BMDx2 through three distinct case studies designed to demonstrate its flexibility in handling diverse data types and analytical configurations. The first case study involves multi-omics data, specifically DNA microarray and DNA methylation, obtained from THP-1 cell lines exposed to rigid carbon nanotubes. The second case study focuses on RNA-Seq data from THP-1 cells treated with bleomycin. The third utilizes cytokine expression and qPCR data, also related to bleomycin exposure. These case studies also exemplify the adaptability of BMDx2 in supporting various model fitting strategies. One approach, applied in the bleomycin case study, involves selecting the best-fitting model based on the AIC. An alternative strategy, employed in the rigid carbon nanotubes case study, is model averaging, which integrates multiple models into a single, to obtain a consensus POD estimate. Both approaches are recognized and recommended in regulatory guidance. The use of the AIC has been endorsed by the European Food Safety Authority (EFSA) as the preferred method for model comparison over the previous log-likelihood approach.^[7]^ In contrast, when a stronger emphasis on predictive uncertainty is warranted, model averaging provides a robust alternative by integrating benchmark dose estimates from multiple models into a single, uncertainty-weighted outcome.^[8]^ Together, these examples underscore the methodological robustness and regulatory relevance of BMDx2 in benchmark dose modeling across a range of experimental contexts.

## **BMDx Analysis of multi-omics gene expression and DNA methylation data** **for rCNT exposure**

#### **Multi-omics rCNT data collection**

The transcriptomics and DNA methylation datasets comprise microarray and genome-wide methylation data from macrophages exposed to rigid multi-walled carbon nanotubes (rCNTs) at three doses (5, 10, and 20 µg/mL) and three consecutive time points (24, 48, and 72 hours). These datasets originate from our previous study and are publicly available at GEO under accession GSE146710.^[32]^ Preprocessing and differential analysis procedures were directly adopted from the previous study without modification.^[32]^ Briefly, the preprocessing and analysis were performed using the Eutopia shiny app.^[33]^ For transcriptomic data, differential expression between exposure and control groups was assessed through linear modeling complemented by empirical Bayes statistics as implemented in the *limma* package incorporating corrected batch effects as covariates.^[2]^ Genes were designated as significantly differentially expressed if they exhibited fold changes exceeding |1.5| and possessed Benjamini-Hochberg adjusted p-values below 0.05. Similarly, the methylation data as preprocessed and analyzed in the previous study were used. Gene promoter regions were specifically delineated as the 200 bp regions upstream of transcription start sites. Median M-values of CpG probes localized within these promoters were calculated and subsequently converted into Beta-values using the lumi R package.^[34]^ Differential methylation analyses were performed with the limma package, identifying significantly altered promoters based on fold changes greater than |1.2| and p-values lower than 0.01.^[2]^ In this work, the union of all the differentially expressed genes from the transcriptomic study, and the genes whose promoter are differentially methylated in the methylation study, were used as a starting point to perform dose dependent analysis.

#### **BMDx analysis of rCTN data**

The BMDx package was used to perform dose dependent analysis of the gene expression and DNA methylation data. First the data were loaded into R with the function *“read_excel_allsheets”*, then they were converted into a list using the function “*create_data_structure*”. Since the doses tested in the study are not equally spaced on a linear scale as for the bleomycin case study, a log transformation to the doses was applied before BMD modelling. Log transformation makes the concentrations evenly spaced, making the relationship easier to model and avoids over-weighting the higher doses. No filtering was performed with the BMDx package since the selection of genes/promoter region taken a priori from the original study.

Afterwards the function “*build_models*” was used to select the models to be fitted. Dose response models including linear, second-degree polynomial (poly2), hill, exponential (exp2) and power models were fitted to all the genes and gene promoter regions of the transcriptomics and DNA methylation data by means of the “*fitting_list*” function. BMD modelling was performed under assumption of constant variance. The BMR factor parameter was set to 1.349, representing a relevant biological change,^[35]^ and a 95% confidence interval was used. After fitting, the *“model_filtering”* function was used to filter out models. Models were excluded if they exhibited an R² value below 0.6 or failed to provide estimates for BMD, BMDL, and BMDU. The choice of R² < 0.6 as a threshold for model exclusion ensured retention of models explaining a substantial portion of variance, minimizing inclusion of poorly fitting models and enhancing the biological interpretability of the results. For all the genes, where multiple models were still present after the modelling filtering, the optimal model was built as the average of all the models, by means of the function “*add_average_models”*. The result is a list of dose dependent genes for each omics layer.    Finally, the optimal model was selected by means of the function “*select_optimal_models*”, with parameter *method = “Model Average”* to specify that the model average should be selected when more than one model is fitted for each gene. Otherwise, for genes, where only one model was available, that was considered the optimal model. Moreover, the statistics for each gene and gene promoter regions for any list of fitted models can be computed by means of the function “*compute_model_statistics*”.

Subsequently, to assess the similarity between gene expression and methylation patterns across doses, we used the function “*gene_pairs_comparison*” that compute the Pearson Correlation between the vectors of the predictions. The function takes in input the list of optimal models for the gene expression, the list of optimal models for the methylation, and a vector of doses that will be given in input to the optimal models to perform their predictions. Such predictions from the optimal model are the vectors between which the function compute the Pearson Correlation and the Euclidean distance. For our case study, we selected 1000 uniformly distributed doses in the 0 to the maximum tested dose. We repeated this comparative analysis three times, each time feeding to the “*gene_pairs_comparison*” function the models fitted at the individual time points.

The “*gene_pairs_comparison*” function gives in output a dataframe that combine the statistics related to model fitting and BMD modelling of the pairs of genes and promoter regions compared along with their correlation values. These dataframes were investigated to identify a core set of genes that resulted as dose dependent in both omics layers. These genes were the divided into two groups based on their sensitivity on the BMD values across the two omics layers. If a gene had a lower BMD in the transcriptomic layer, it was marked as more sensitive in transcriptomic, otherwise as more sensitive in methylation. The “*gost*” function from the gprofiler2 package^[27]^ was used to perform an enrichment analysis of the two list of genes.

Finally, the core set of genes with consistent dose-dependent profile across the two omics layers was further investigated with respect to their dose-dependent behavior across the three time points. The “*xtabs*” function R function was used to identify the binary activation profiles of the genes in each of the two omics. The relevant combinations described in Figure 3 were retrieved and the “*pheatmap*” and *ComplexHeatmap* R libraries were used for visualization purposes. Finally, the “*enrich_KEs_AOPs*” function from the AOPfingeprintR package was used to annotate the genes genes from the different combinations to KEs. All the scripts to reproduce the analysis are available at: <https://doi.org/10.5281/zenodo.16992577>. The description of the BMDx functions can be found in the manual <https://github.com/fhaive/bmdx/blob/main/manual.pdf>.

## **BMDx2 analysis of RNASeq analysis for bleomycin exposure**

### **RNASeq bleomycin data collection**

The transcriptomics dataset comprises RNA-Seq data from bleomycin exposure at six doses (0, 20, 40, 60, 80, 100 µg/mL) and three time points (24, 48, and 72 hours), with four biological replicates at each dose-time combination.^[36]^ The inclusion of four replicates per condition is a critical feature, as it enhances the statistical power of the analysis, reduces variability, and increases the reliability of detecting dose-dependent changes in gene expression. Additionally, the use of equidistant doses ensures a uniform distribution across the dose range, minimizing the need for model interpolation. This avoids potential biases or inaccuracies that can arise when doses are unevenly spaced or cover a wide range, leading to more robust and interpretable dose-response relationships.  RNA extraction, sequencing and analysis is described in the original publication.^[36]^ Briefly, the analysis followed these steps: the raw sequencing reads underwent an initial quality assessment using FastQC v0.11.7 (<https://github.com/s-andrews/FastQC/blob/master/RELEASE_NOTES.txt>), followed by the trimming of Illumina adapters and removal of low-quality bases with TrimGalore v0.4.4_dev (<https://github.com/FelixKrueger/TrimGalore>). After trimming, quality control was reassessed using FastQC v0.11.7. The processed reads were then aligned to the human reference genome (GRCh38) using HISAT2 v2.1.0.^[37]^ Following alignment, BAM files were indexed, and uniquely mapped reads were generated with SAMtools v1.827-g0896262.^[38]^ Gene-level read counts were quantified using the featureCounts function from the Rsubread package v1.34.6.^[39]^ Low-expressed genes were filtered out using a proportion test, as implemented in the R package NOIseq v2.28.0.^[40]^ For more details, please refer to Morikka et al.^[36]^ Finally the raw counts were normalized with the variance-stabilized transformation (VST)^[41]^ implemented in DESeq2. Normalization and variance stabilization are crucial for RNA-Seq data in BMD analysis, as raw data often violates the homoscedasticity assumption. VST addresses this by reducing the dependence of variance on mean expression levels, thus improving result accuracy for large datasets (Figure S 13).

### **BMDx2 analysis of bleomycin data**

**Data processing and filtering**

First the phenotype data and VST normalized count expression data were imported into the BMDx2 app. A pre-filtering step was applied using differential expression analysis. Genes exhibiting significant variation across experimental conditions were retained, defined as those with an adjusted p-value < 0.05 and an absolute fold change (FC) >1.5 relative to controls.

**BMD modelling**

For each selected gene, dose-response relationships were modeled using power, hill, polynomial (degree 2), and linear regression models, assuming constant variance. A BMR factor of 1.349 was applied, it signifies a 10% increase in biological responses in the treated group over the control's baseline, a standard threshold for defining a meaningful effect in BMD analysis.^[5]^ Model fitting was assessed using R². Models with R² values <0.6, or those failing to yield estimates for the BMDL, BMD, and BMDU, were excluded. This filtering step ensured the retention of only well-fitted models with biologically plausible parameter estimates, enhancing the robustness and interpretability of the results. The optimal model for each gene was selected based on the lowest AIC value.

**Downstream analysis**

The FunMappOne section of the BMDx2 app was used to perform and plot the comparative KEGG enrichment analysis for the DDGs identified at the three time points (24, 48 and 72 hours).

In addition, genes were ranked based on their frequency of dose-dependence across time points, thereby prioritizing genes with consistent dose-response patterns. To identify enriched biological pathways and functions associated with most frequent dose-responsive genes, gene ontology and pathway enrichment analysis was performed using the *gProfiler* package *gost* function (Figure S 15).

To further explore the relationships between dose-responsive genes at the 72-hour time point, a gene-pair analysis was conducted. This analysis compared the predicted dose-response trajectories for each gene pair, by computing the Pearson correlation between these trajectories. Genes were subsequently grouped with the hierarchical clustering algorithm and 3 sets were identified, For clusters containing at least 20 genes, enrichment analysis was performed using the “*gost”* function from the *gProfiler* package to identify enriched biological pathways (Figure S 17).

Furthermore, the DDGs identified at each time point were subsequently annotated to AOPs. Following the methodology outlined in Saarimaki et al.^[21]^, implemented in the AOPfingerprintR package, AOP fingerprints were generated for each time point. Specifically, AOPs with at least 5 KEs were considered significantly enriched if at least 33% of their KEs were enriched (Figure S 17). Moreover, a KE-KE network was built (Figure 5), based on the enriched KEs at 72 hours, to visualize and explore potential interconnections and dependencies between these pathways. The network included all enriched KEs and was extended by integrating intermediate KEs required to connect them to a maximum of two MIEs and two AOs per enriched KE, within a maximum path length of three steps.

Subsequently, the twPOD was calculated for each time point using various methods, including the mean, percentile, first mode, lowest, and accumulation, considering the set of dose-responsive genes identified at each time point (Figure S 19).

The data and log file from the BMDx2 app to reproduce the analysis are available at: <https://doi.org/10.5281/zenodo.16992577>.

### **Results RNASeq bleomycin**

#### **Characterization of the dose-response mechanism of action of chemical exposure though KEGG enrichment analysis**

To investigate the functions underscoring these genes, KEGG pathways enrichment analysis of dose-dependent genes was performed in the FunMappOne section of the tool. At 24 hours, TNF and NF-κB signaling pathways are enriched at low BMD (Figure S 14A), aligning with acute inflammation.^[36,42]^ By 72 hours NF-κB is enriched with a higher BMD, suggesting an adaptive dynamic dose-dependent response. The consistent enrichment of the Cytokine-cytokine receptor interaction pathway underscores the central role of cytokine signaling.^[43]^ The tool also allows to investigate the genes driving this enrichment and their estimated BMD values. This analysis revealed that the dose-dependent contributions vary across time points (Figure S 14B), suggesting a dynamic adaptation of cytokine signaling, transitioning from acute inflammation genes at 24 hours to those involved in resolution and repair at the later 48 hours and 72 hours time points. Although bleomycin appears to affect a larger number of genes at the latter time points, their increased BMD values compared to 24 hours of continuous exposure suggests a potential decrease in the chemical’s potency despite continued pathway activity. These observations would not be apparent without the application of BMD modelling highlighting the informative nature of this approach.

The tool enables comparison of gene activation across conditions. Here we compared the genes across the time points (Figure S 14 C). Although the total number of dose-dependent genes at 48 and 72 hours is similar, many are unique to each time point, with only 72 genes shared across all three (Figure S 14 C). These genes may reflect the most persistent dose-responsive effects of bleomycin. Consequently, an enrichment analysis was performed on the most frequent dose dependent genes (Figure S 15, Table S9). The data indicate that the most frequently observed dose-dependent genes are central to both cell growth regulation and inflammatory signaling, revealing the primary impact of the chemical on cellular proliferation and immune response. This aligns with the established understanding of the toxicity of bleomycin, which involves DNA damage leading to cell death and the activation of inflammatory pathways.^[44]^ The consistent identification of these processes among the most frequently observed dose dependent genes underscores their strong responsiveness to bleomycin exposure and proves the effectiveness of the BMDx2 tool.

Further relevant information provided by the BMDx2 tool, is the direction of the fitted model, indicating increasing or decreasing expression values across the doses. This can give valuable insights into how gene expression changes with increasing bleomycin exposure concentration. Figure S 14 D demonstrates an increasing trend in the number of genes with an "increasing" (+1) model direction over time, suggesting that prolonged bleomycin exposure leads to an upregulation of a larger number of genes as the dose increases.

## **Case study Targeted *In Vitro* Data:**

## **Case study on the Dose-Dependent Mechanisms of Action (MOA) Using Targeted *In Vitro* Data**

This section shows the application of our BMD analysis tool to qPCR and immunoassay data, expanding its utility beyond traditional transcriptomic analyses to provide a more comprehensive understanding of chemical toxicity. qPCR provides targeted and quantitative measurement of gene expression for specific genes of interest, while immunoassays enable direct quantification of protein levels, especially for secreted proteins such as cytokines, crucial mediators of cellular communication and often key endpoints in toxicity studies. With the same experimental design of the previous case study (THP1 exposed to bleomycin multiple doses and time points), Morikka et al. also produced qPCR and immunoassay data. qPCR targeted identified a panel of immune-related genes, while the ProcartaPlex 22-plex immunoassay quantified a panel of immune cytokines.^[36]^ We used the BMDx package to perform the dose dependent analysis of such datasets.

### **Dataset**

qPCR and immunoassay data were collected from Morikka et al. ^[36]^ In the qPCR experiment, THP1 cells were exposed to bleomycin at four concentrations 0, 20, 80, and 100 µg/ml for 24,48 and 72 hours. Conversely, the immunoassay experiment employed six bleomycin exposure concentrations 0, 20, 40, 60, 80, and 100 µg/ml for 24, 48and 72 hours. For the qPCR and Immunoassay data, features (e.g. gene for qPCR and protein for Immune assay) exhibiting missing values (NAs) at specific time points were excluded from analysis at those time points, ensuring only complete data were used for dose-response modeling.

### **Analysis**

For each retained feature, dose-response relationships were modeled assuming constant variance. Due to the higher dose range in the immunoassay dataset, a broader selection of models was employed, including linear, polynomial (degrees 2-5), Hill, power, and exponential (degrees 2-5) models. In contrast, the qPCR dataset utilized linear, polynomial (degree 2), Hill, exponential (degree 2), and power models. The function “*build_models*” was used to select the models to be fitted, and the data were fitted by means of the “*fitting_list*” function. A BMR factor of 1.349, representing a biologically relevant change, was applied to all models. Model R² < 0.6 or those failing to generate estimates for the BMDL, BMD and BMDU were discarded with the *“model_filtering”* function. The optimal model for each feature was selected with “*select_optimal_models*” function, with parameter *method = “AIC”*; for genes, where only one model was available, that was considered the optimal model. The statistics for each gene and gene promoter regions for any list of fitted models can be computed by means of the function “*compute_model_statistics*”. Following pathway enrichment analysis of dose-dependent qPCR genes with FunMappone, the average BMD range (BMDL to BMDU) for genes within each enriched term was graphically represented, facilitating the comparison of dose-response sensitivities across different biological pathways. The code and data used for this analysis can be found at <https://doi.org/10.5281/zenodo.16992577>. The description of the BMDx functions can be found in the manual <https://github.com/fhaive/bmdx/blob/main/manual.pdf>.

### **Results**

#### **Dose-dependent analysis of Cytokines**

Analysis of the cytokines revealed that the number of dose-dependent cytokines varied over time: 6 at 24 hours, 9 at 48 hours, and 5 at 72 hours (Figure S 21 and Table S 11). The early time point at 24 hours is characterized by the presence of IL-1A, IL-1B, and IL-18, suggesting an immediate pro-inflammatory response. These cytokines are key mediators of innate immunity and are known to initiate inflammation by promoting the activation of immune cells and amplifying cytokine signaling.^[45]^ The relatively low BMD values associated with these cytokines indicate that they respond to even minimal perturbations, reinforcing their role as early sentinels in the immune cascade. At 48 hours, the immune response undergoes a shift, with IL-10, a well-established anti-inflammatory cytokine, exhibiting the highest BMD value, and its presence at this time point suggests a counter-regulatory mechanism aimed at dampening excessive inflammation.^[46]^ The transient appearance of IL-17A shows a brief activation of the Th17 pathway, which is well known to be involved in chronic inflammation and fibrosis.^[47]^ These two events suggest that by 48 hours, the immune system perceives ongoing bleomycin exposure as progressing from acute to chronic inflammation. Continued FGF2 and CCL2 expression at 72 hours signals a transition to tissue repair and possible fibrosis. Specifically, FGF2 sustains fibroblast activity, and CCL2 maintains immune cell recruitment, which demonstrates a low BMD, suggesting decreased sensitivity to dose changes over time.^[48,49]^ The consistent BMD value of MMP1 across all time points, indicating its persistent detection, suggests continuous ECM remodeling independent of bleomycin concentration, reflecting typical tissue injury and repair processes.^[50]^

#### **Dose-dependent analysis of qPCR**

The benchmark dose analysis from qPCR gene shows 10, 17 and 12 DDGs respectively at 24, 48 and 72 hours (Figure S 22A and Table S 12). To gain insights into the biological functions and pathway contexts of these genes, particularly in relation to their sensitivity to bleomycin, we explored their mapping across relevant biological pathways. Rather than focusing on statistical overrepresentation, this analysis aimed to identify the functional landscape and molecular processes associated with the DDGs at each time point. BMDx2 also allows to represent the range plot of the BMD values of the genes associated to specific pathways (Figure S 22B and Table S 12). Such analysis reveals a trend consistent with our RNA-seq data: a decrease in bleomycin sensitivity at later time points (72 hours), evidenced by a wider BMD range. This suggests a potential adaptation or resistance mechanism developing over time. Notably, chemokine signaling pathways remained consistently enriched across all time points, reinforcing our earlier observation of their sustained activity in response to bleomycin. Furthermore, the 24 hours time point showed enrichment in acute inflammatory pathways, such as TNF and NOD-like receptor signaling, which were less prominent at later time points. This suggests an early, robust inflammatory response that diminishes as the cells transition to other regulatory or reparative processes. This analysis highlights the power of BMD approaches applied to qPCR and immunoassay data, extending its utility beyond transcriptomics for a more comprehensive chemical safety assessment.

# **Supplementary Figures**

Figure S 1: Components of the BMDx2 suite. The Shiny APP is based on three R packages (BMDx, AOP Fingerprint and FunMappOne). The BMDx and AOP Fingerprint package functionalities are also offered through REST APIs.

Figure S 2: Pipeline implemented in BMDx for dose-dependent analysis.

Figure S 3: BMD modelling strategy. For each fitted model, the BMR can be estimated (A) through three different strategies:1) relative method, 2) absolute method and 3) standard deviation method. The standard deviation method further depends on the variance assumption. Three variance assumption strategies can be modelled in BMDx2: 1) constant variance, 2) parametric and 3) nonparametric variance. Once the BMR is estimated, this can be used to estimate the BMD (B), finally, the lower and upper bounds (BMDL and BMDU) are estimated though the Wald method.

Figure S 4: Number of genes in KEs in the original mapping from Saarimäki et al^[21]^ (Human) to Mouse and Rat genes. This plot considers the Ensemble gene IDs. The lower panels are a zoom of the above ones, where outliers have been removed

Figure S 5:Number of AOP associated to one or two SSbD Category, or uncategorized.

Figure S 6: Number of AOPs for each SSbD category and for each organ.

Figure S 7: Number of AOPs for each SSbD category and for each endpoint

Figure S 8: Venn diagram of the number of dose dependent genes across the three time points (A); Venn diagram of the number of dose-dependent methylated promoter regions across the three time points (B). Venn diagram of the dose dependent genes and dose-dependent

Figure S 9: Distribution of BMD values in gene expression at 24, 48 and 72 hours (A-B); Distribution of BMD values in DNA methylation at 24, 48 and 72 hours (C-D).

Figure S 10: Number of sensitive genes in gene expression and methylation data

Figure S 11: Heatmap illustrating the enrichment of Key Events (KEs) by gene groups categorized according to their dose-dependent transcriptional (GEX) and epigenetic (MET) response profiles across 24, 48, and 72 hours. Each column represents a distinct gene group. Gene annotation to KE performed with Fisher test. Nominal p-value < 0.05. N dose-dependent genes (Sustained GEX & Late MET (S-L) = 79, Sustained GEX & Mid & Late MET (S-ML) = 6, Sustained GEX & Sustained MET (S-S) = 2, Mid-Late GEX & Late MET (ML-L) = 27, Mid-Late GEX & Early MET (ML-E)=15, Late GEX & Late MET (L-L)=16, Late GEX & Early MET (L-E) = 11, Late GEX & Early-Mid MET (L-EM) = 1.

Figure S 12: Pearson correlation analysis (N=1000) between gene profiles at 24 hours in transcriptomics and the same gene promoters at 72 hours. Direction of gene expression and DNA methylation profiles is also reported.

Figure S 13: (A) relationship between expression values and standard deviation on raw count data. (B) relationship between expression value and standard deviation after VST transformation.

Figure S 14: Dose dependent MOA characterization. (A) The KEGG pathways significantly enriched by the dose-dependent genes at the three time points, colored by average BMD value. Enriched terms were identified with Fisher's one-tailed test and the significancy is based on a Set Counts and Sizes-adjusted p-value threshold of < 0.05. (B) The dose dependent genes of the Cytokine-cytokine receptor interaction pathway at the three time

Figure S 15: GSEA enrichment analysis performed on top of the dose-dependent genes ranked based on their frequency of dose-dependent behavior across the three time points. Enrichment has been tested with Fisher's one-tailed test, and significantly enriched terms were identified based on an FDR-adjusted p-value threshold of < 0.05.

Figure S 16: Enrichment analysis of the gene co-expression cluster analysis of the dose-dependent genes after bleomycin exposure in THP-1 at 72 hours. Enrichment has been tested with Fisher's one-tailed test, and significantly enriched terms were identified based on an FDR-adjusted p-value threshold of < 0.05.

Figure S 17: Bubble plot of AOP fingerprint for multiple time points. Each bubble represents an AOP, its size reflects the proportion of enriched KEs, and the color indicates enrichment strength. Enriched terms were identified with Fisher's one-tailed test and significantly enriched terms were identified based on Fisher test, FDR-adjusted p-value threshold of < 0.05.

Figure S 18: Density distribution of the transcriptome wide BMD. (B) ECDF plot of the transcriptome wide BMD.

Figure S 19: Transcription wide Point Of Departure (twPOD) computed with lowest LCRD, Percentile (20), First Mode and Accumulation Plot Maximum Curvature method at 24h (A), 48h (B) and 72h (C). The x-axis has been limited to 1 to appreciate better the twPOD estimate.


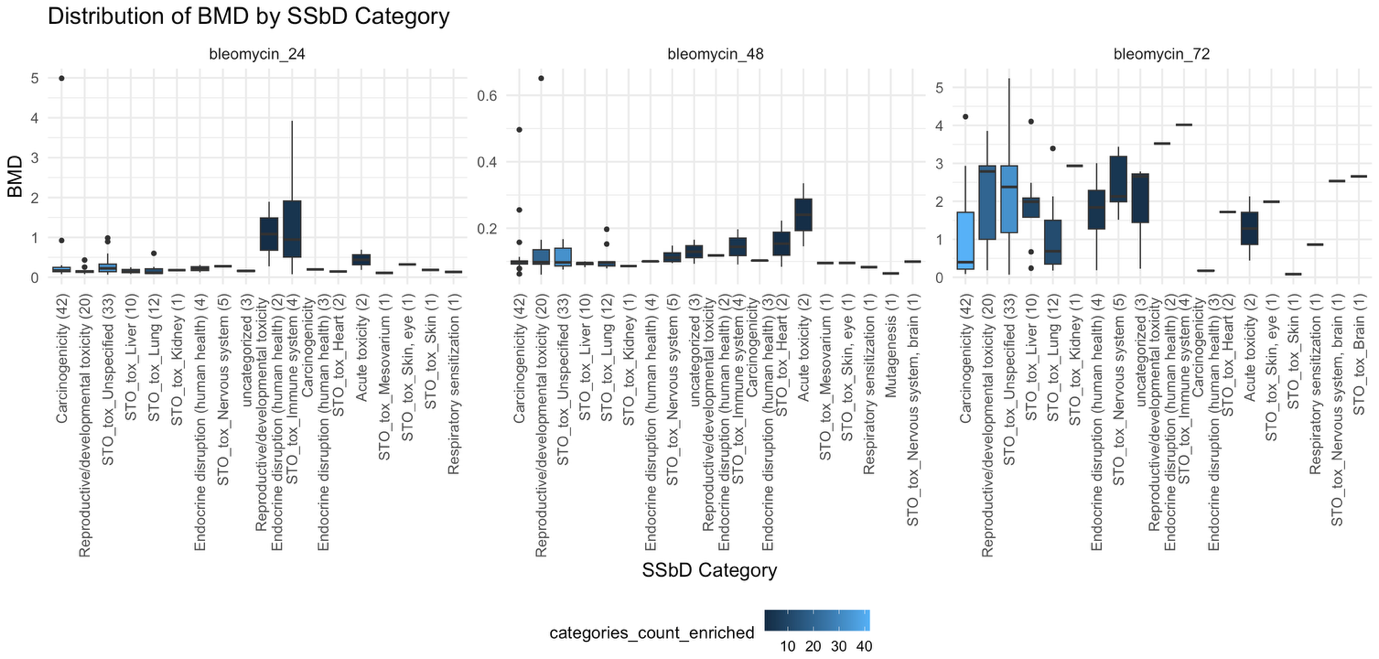


Figure S 20: Distribution of BMD values in AOPs grouped for each hazard class related to human health suggested by JRC for the SSbD framework. STO_tox stands for specific target organ toxicity. Number in brackets next to the hazard classes represent the number of AO.

Figure S 21: Heatmap showing the BMD values of selected immune-related proteins across the time points.

Figure S 22: (A) Heatmap showing the BMD values of selected target from qPCR analysis across the time points. (B) Range plots depicting the BMD values for genes within enriched pathways across the same time points. Enriched terms were identified with Fisher's one-tailed test and the significancy is based on a Set Counts and Sizes-adjusted p-value threshold of < 0.05. Each plot shows the BMD range (BMDL to BMDU) for individual genes within the indicated pathways. The red, blue, and green points represent the BMDL, BMDU, and BMD values, respectively, providing a visual representation of the variability in gene sensitivity to dose within these enriched pathways.

# **Supplementary Tables**

Table S 1: list of dose-response models available in BMDx2

| **Model** | **Formula** |
| --- | --- |
| **Linear** | f(dose) = β₀ + β₁ × dose  Where β₀ is the control response (intercept), and β₁ is the slope. |
| **polynomial models** | f(dose) = β₀ + β₁ × dose + β₂ × dose² + … + βₙ × doseⁿ  Where β₀ is the control response (intercept), β₁ to βₙ are the polynomial coefficients, and n is the degree of the polynomial. |
| **Power** | f(dose) = β₀ + β₁ × (dose)ᵟ  Where β₀ is the control response (intercept), β₁ is the slope, and δ is the power. |
| **Hill** | f(dose) = β₀ + (v × doseⁿ) / (Kⁿ + doseⁿ)  Where β₀ is the control response, v is the maximum response, K is the dose at which half the maximum response is reached, and n is the Hill coefficient. |
| **Exponential** | Exp2: f(dose) = a × e^(± b × dose)  Exp3: f(dose) = a × e^(± (b × dose)ᵈ)  Exp4: f(dose) = a × (c - (c - 1) × e^(± b × dose))  Exp5: f(dose) = a × (c - (c - 1) × e^(± (b × dose)ᵈ))  Where a is the control response (intercept), b is the slope, c is the asymptote term, and d is the power. |
| **Log-Logistic Model** | Log-Logistic 5:  f(dose) = c + (d - c) / (1 + exp(b × (log(dose) - log(e))))ᶠ  If f ≠ 1, the function is asymmetric; otherwise, it is symmetric (on a log scale).  Log-Logistic 4:  f(dose) = c + (d - c) / (1 + exp(b × (log(dose) - log(e))))  The function is symmetric about the inflection point e.  Log-Logistic 3:  f(dose) = c + (1 - c) / (1 + exp(b × (log(dose) - log(e))))  The function is symmetric about the inflection point e.  Log-Logistic 2:  f(dose) = 1 / (1 + exp(b × (log(dose) - log(e))))  The function is symmetric about the inflection point e. |
| **Michaelis-Menten Model** | MM.3 Model:  f(dose) = c + (d - c) / (1 + (e / dose))  This model increases as a function of dose, attaining the lower limit c at dose 0 and the upper limit d for infinitely large doses. The parameter e corresponds to the dose yielding a response halfway between c and d.  MM.2 Model:  A two-parameter version of the Michaelis-Menten model is obtained by setting c = 0. |
| **Weibull** | Weibull 1.2:  f(dose) = 1 - exp(-b × doseᵉ)  This model describes an asymmetric sigmoidal growth curve.  Weibull 1.3:  f(dose) = d - d × exp(-b × doseᵉ)  Extends Weibull 1.2 by adding a scaling parameter d that adjusts the upper asymptote.  Weibull 1.4:  f(dose) = c + (d - c) × (1 - exp(-b × doseᵉ))  Allows both upper (d) and lower (c) asymptotes, providing greater flexibility.  Weibull 2.2:  f(dose) = exp(-exp(b × (log(dose) - e)))  A sigmoidal decay function where e is the inflection point.  Weibull 2.3:  f(dose) = d × exp(-exp(b × (log(dose) - e)))  Extends Weibull 2.2 with a scaling parameter d, modifying the maximum response.  Weibull 2.4:  f(dose) = c + (d - c) × exp(-exp(b × (log(dose) - e)))  Generalizes Weibull 2.3 by incorporating both lower (c) and upper (d) asymptotes. |
| **Average Model** | $\frac{\exp\left( -\frac{AIC_{i}-AIC_{min}}{2} \right)}{\sum\exp\left( -\frac{AIC_{i}-AIC_{min}}{2} \right)}$  where $w_{i}$ represents the relative weight of model $i$ |

Table S 2: Comparison with other tools

|  |  | BMDExpress2 | BMDx2 | DROmics | FastBMD | BBMD | BMDS |
| --- | --- | --- | --- | --- | --- | --- | --- |
| Implementation | Reference |  |  |  |  |  |  |
|  | Platform | Standalone | R Shiny  R package and API for the BMDx and AOPFingerprint packages | R package  R Shiny web app | Web app | Web app | Standalone |
|  | Open source | Yes | Yes | Yes | No | No |  |
|  | Programming language | Java | R | R | R  JavaServer Faces (JSF) | Python  Javascript |  |
| Data types | Types of data | Transcriptomics data (does not differentiate microarray and RNAseq data) or other continuous data | Continuous omic data (microarray, normalized RNASeq) | RNAseq, microarray, continuous omics data (e.g. metabolomics, proteomics), continuous anchoring data | RNAseq, microarray | RNAseq, microarray,  continuous anchoring data, binary anchoring data |  |
|  | Visualization input data | PCA + Density | PCA |  |  |  |  |
|  | Filtering |  | Anova  Trend  Fold Change |  |  |  |  |
|  | Compare multiple experiments | Yes | Yes | No | No |  | No |
|  | Comparative visualization | Yes | Yes | No | Yes |  | No |
| BMD modelling | Available models | Linear Polynomial Hill  Power Exponential | Linear Polynomial  Hill Power Exponential  Log-logistic* Weibull*  Brain-Counsen* Asymptotic* Michaelis-Mentel* | Linear  Hill Exponential Gauss-probit Log-Gauss-probit | Linear Polynomial Hill  Power Exponential |  | Linear Polynomial  Hill  Power Exponential |
|  | Model avarage |  | Yes |  |  |  |  |
|  | Best selection of models |  | AIC  Average (user choice) |  |  |  |  |
|  | Effective doses |  | BMD  BMDL BMDU  IC50 |  |  |  |  |
|  | Confidence interval |  |  |  |  |  |  |
| Biological interpretation | Functional annotation | Yes | Yes | No | Yes | Yes |  |
|  | Annotation type |  | KEGG, Reactome,  GO |  |  |  |  |
|  | Organisms |  | Homosapiens, Musmusculus, Rattusnorvegicus |  |  |  |  |
|  | AOP enrichment | No | Yes | No | No | No | No |
| Gene co-expression analysis |  |  | Yes |  |  |  |  |

Table S3: Dose dependent genes identified at the transcriptomic level for rCNT exposure in THP-1

Table S4: Dose dependent gene promoter regions identified at the DNA methylation level for rCNT exposure in THP-1

Table S5: Genes more sensitive in DNA methylation for rCNT exposure in THP-1

Table S6: Genes more sensitive in transcriptomics for rCNT exposure in THP-1

Table S7: Enrichment analysis on the set of sensitive genes for rCNT exposure in THP-1

Table S8: Dose dependent genes identified at the transcriptomic level for bleomycin exposure in THP-1

Table S9: GSEA enrichment analysis on the dose-dependent genes altered in THP-1 after bleomycin exposure ordered based on their frequency across the time points. FDR-adjusted pvalues.

Table S10: Clustering analysis based on gene co-dose dependency analysis for bleomycin exposure at 72 hours.

Table S11: Dose dependent cytokines identified from immune assay data for bleomycin exposure in THP-1.

Table S12: Dose dependent genes base on qPCR data for bleomycin exposure in THP-1.

Table S13: Enrichment analysis on the set of qPCR-derived sensitive genes for bleomycin exposure in THP-1.

# **References**

1. Schloerke B, Allen J. plumber: An API Generator for R [Internet]. 2025. Available from: https://www.rplumber.io

2. Ritchie ME, Phipson B, Wu D, Hu Y, Law CW, Shi W, et al. limma powers differential expression analyses for RNA-sequencing and microarray studies. Nucleic Acids Res 2015;43(7):e47.

3. Elzhov TV, Mullen KM, Spiess AN, Bolker B. minpack.lm: R Interface to the Levenberg-Marquardt Nonlinear Least-Squares Algorithm Found in MINPACK, Plus Support for Bounds [Internet]. 2023 [cited 2025 Mar 26];Available from: https://cran.r-project.org/web/packages/minpack.lm/index.html

4. Ritz C, Strebig JC. drc: Analysis of Dose-Response Curves [Internet]. 2016 [cited 2025 Mar 26];Available from: https://cran.r-project.org/web/packages/drc/index.html

5. Thomas RS, Allen BC, Nong A, Yang L, Bermudez E, Clewell HJ, et al. A Method to Integrate Benchmark Dose Estimates with Genomic Data to Assess the Functional Effects of Chemical Exposure. Toxicol Sci 2007;98(1):240–8.

6. Gaylor D, Ryan L, Krewski D, Zhu Y. Procedures for calculating benchmark doses for health risk assessment. Regul Toxicol Pharmacol RTP 1998;28(2):150–64.

7. Committee ES, Hardy A, Benford D, Halldorsson T, Jeger MJ, Knutsen KH, et al. Update: use of the benchmark dose approach in risk assessment. EFSA J 2017;15(1):e04658.

8. Committee ES, More SJ, Bampidis V, Benford D, Bragard C, Halldorsson TI, et al. Guidance on the use of the benchmark dose approach in risk assessment. EFSA J 2022;20(10):e07584.

9. Satopaa V, Albrecht J, Irwin D, Raghavan B. Finding a ‘Kneedle’ in a Haystack: Detecting Knee Points in System Behavior [Internet]. In: 2011 31st International Conference on Distributed Computing Systems Workshops. 2011 [cited 2025 Mar 28]. page 166–71.Available from: https://ieeexplore.ieee.org/abstract/document/5961514

10. Pagé-Larivière F, Crump D, O’Brien JM. Transcriptomic points-of-departure from short-term exposure studies are protective of chronic effects for fish exposed to estrogenic chemicals. Toxicol Appl Pharmacol 2019;378:114634.

11. Farmahin R, Williams A, Kuo B, Chepelev NL, Thomas RS, Barton-Maclaren TS, et al. Recommended approaches in the application of toxicogenomics to derive points of departure for chemical risk assessment. Arch Toxicol 2017;91(5):2045–65.

12. Costa E, Johnson KJ, Walker CA, O’Brien JM. Transcriptomic point of departure determination: a comparison of distribution-based and gene set-based approaches. Front Genet [Internet] 2024 [cited 2025 Mar 28];15. Available from: https://www.frontiersin.org/journals/genetics/articles/10.3389/fgene.2024.1374791/full

13. gnls: Fit Nonlinear Model Using Generalized Least Squares in nlme: Linear and Nonlinear Mixed Effects Models [Internet]. [cited 2025 Mar 27];Available from: https://rdrr.io/cran/nlme/man/gnls.html

14. Symonds MRE, Moussalli A. A brief guide to model selection, multimodel inference and model averaging in behavioural ecology using Akaike’s information criterion. Behav Ecol Sociobiol 2011;65(1):13–21.

15. Reardon AJF, Farmahin R, Williams A, Meier MJ, Addicks GC, Yauk CL, et al. From vision toward best practices: Evaluating in vitro transcriptomic points of departure for application in risk assessment using a uniform workflow. Front Toxicol [Internet] 2023 [cited 2025 Apr 23];5. Available from: https://www.frontiersin.orghttps://www.frontiersin.org/journals/toxicology/articles/10.3389/ftox.2023.1194895/full

16. Matteo G, Leingartner K, Rowan-Carroll A, Meier M, Williams A, Beal MA, et al. In vitro transcriptomic analyses reveal pathway perturbations, estrogenic activities, and potencies of data-poor BPA alternative chemicals. Toxicol Sci 2023;191(2):266–75.

17. Crizer DM, Ramaiahgari SC, Ferguson SS, Rice JR, Dunlap PE, Sipes NS, et al. Benchmark Concentrations for Untargeted Metabolomics Versus Transcriptomics for Liver Injury Compounds in In Vitro Liver Models. Toxicol Sci 2021;181(2):175–86.

18. Qutob SS, Chauhan V, Kuo B, Williams A, Yauk CL, McNamee JP, et al. The application of transcriptional benchmark dose modeling for deriving thresholds of effects associated with solar-simulated ultraviolet radiation exposure. Environ Mol Mutagen 2018;59(6):502–15.

19. Johnson KJ, Costa E, Marshall V, Sriram S, Venkatraman A, Stebbins K, et al. A microRNA or messenger RNA point of departure estimates an apical endpoint point of departure in a rat developmental toxicity model. Birth Defects Res 2022;114(11):559–76.

20. Scala G, Serra A, Marwah VS, Saarimäki LA, Greco D. FunMappOne: a tool to hierarchically organize and visually navigate functional gene annotations in multiple experiments. BMC Bioinformatics 2019;20(1):79.

21. Saarimäki LA, Fratello M, Pavel A, Korpilähde S, Leppänen J, Serra A, et al. A curated gene and biological system annotation of adverse outcome pathways related to human health. Sci Data 2023;10(1):409.

22. Saarimäki LA, Morikka J, Pavel A, Korpilähde S, del Giudice G, Federico A, et al. Toxicogenomics Data for Chemical Safety Assessment and Development of New Approach Methodologies: An Adverse Outcome Pathway-Based Approach. Adv Sci 2023;10(2):2203984.

23. Pavel A, del Giudice G, Federico A, Di Lieto A, Kinaret PAS, Serra A, et al. Integrated network analysis reveals new genes suggesting COVID-19 chronic effects and treatment. Brief Bioinform 2021;22(2):1430–41.

24. Caldeira C, Farcal R, Garmendia AI, Mancini L, Tosches D, Amelio A, et al. Safe and sustainable by design chemicals and materials - Framework for the definition of criteria and evaluation procedure for chemicals and materials [Internet]. JRC Publ. Repos.2022 [cited 2025 Mar 26];Available from: https://publications.jrc.ec.europa.eu/repository/handle/JRC128591

25. Patinha CC, Farcal R, Moretti C, Mancini L, Rauscher H, Rasmussen K, et al. Safe and Sustainable by Design chemicals and materials Review of safety and sustainability dimensions, aspects, methods, indicators, and tools [Internet]. JRC Publ. Repos.2022 [cited 2025 Mar 26];Available from: https://publications.jrc.ec.europa.eu/repository/handle/JRC127109

26. Lin S. Space Oriented Rank-Based Data Integration. Stat Appl Genet Mol Biol [Internet] 2010 [cited 2025 Aug 26];9(1). Available from: https://www.degruyterbrill.com/document/doi/10.2202/1544-6115.1534/html

27. Kolberg L, Raudvere U, Kuzmin I, Vilo J, Peterson H. gprofiler2 -- an R package for gene list functional enrichment analysis and namespace conversion toolset g:Profiler. F1000Research 2020;9:ELIXIR-709.

28. Barabási AL, Oltvai ZN. Network biology: understanding the cell’s functional organization. Nat Rev Genet 2004;5(2):101–13.

29. Pavel A, Serra A, Cattelani L, Federico A, Greco D. Network Analysis of Microarray Data [Internet]. In: Agapito G, editor. Microarray Data Analysis. New York, NY: Springer US; 2022 [cited 2025 Mar 27]. page 161–86.Available from: https://doi.org/10.1007/978-1-0716-1839-4_11

30. Durinck S, Moreau Y, Kasprzyk A, Davis S, De Moor B, Brazma A, et al. BioMart and Bioconductor: a powerful link between biological databases and microarray data analysis. Bioinforma Oxf Engl 2005;21(16):3439–40.

31. Csárdi G, Nepusz T, Traag V, Horvát S, Zanini F, Noom D, et al. igraph: Network Analysis and Visualization [Internet]. 2025 [cited 2025 Mar 27];Available from: https://cran.r-project.org/web/packages/igraph/index.html

32. Saarimäki LA, Kinaret PAS, Scala G, del Giudice G, Federico A, Serra A, et al. Toxicogenomics analysis of dynamic dose-response in macrophages highlights molecular alterations relevant for multi-walled carbon nanotube-induced lung fibrosis. NanoImpact 2020;20:100274.

33. Marwah VS, Scala G, Kinaret PAS, Serra A, Alenius H, Fortino V, et al. eUTOPIA: solUTion for Omics data PreprocessIng and Analysis. Source Code Biol Med 2019;14(1):1.

34. Du P, Kibbe WA, Lin SM. lumi: a pipeline for processing Illumina microarray. Bioinformatics 2008;24(13):1547–8.

35. Thomas RS, Allen BC, Nong A, Yang L, Bermudez E, Clewell HJ III, et al. A Method to Integrate Benchmark Dose Estimates with Genomic Data to Assess the Functional Effects of Chemical Exposure. Toxicol Sci 2007;98(1):240–8.

36. Morikka J, Federico A, Möbus L, Inkala S, Pavel A, Sani S, et al. Toxicogenomic assessment of in vitro macrophages exposed to profibrotic challenge reveals a sustained transcriptomic immune signature. Comput Struct Biotechnol J 2024;25:194–204.

37. HISAT: a fast spliced aligner with low memory requirements | Nature Methods [Internet]. [cited 2025 Aug 29];Available from: https://www.nature.com/articles/nmeth.3317

38. Li H, Handsaker B, Wysoker A, Fennell T, Ruan J, Homer N, et al. The Sequence Alignment/Map format and SAMtools. Bioinformatics 2009;25(16):2078–9.

39. Liao Y, Smyth GK, Shi W. featureCounts: an efficient general purpose program for assigning sequence reads to genomic features. Bioinformatics 2014;30(7):923–30.

40. Tarazona S, Furió-Tarí P, Turrà D, Pietro AD, Nueda MJ, Ferrer A, et al. Data quality aware analysis of differential expression in RNA-seq with NOISeq R/Bioc package. Nucleic Acids Res 2015;43(21):e140.

41. Love MI, Huber W, Anders S. Moderated estimation of fold change and dispersion for RNA-seq data with DESeq2. Genome Biol 2014;15(12):550.

42. Meunier É, Aubin vega M, Adam D, Privé A, Mohammad Nezhady MA, Lahaie I, et al. Evaluation of interleukin-1 and interleukin-6 receptor antagonists in a murine model of acute lung injury. Exp Physiol 2024;109(6):966–79.

43. Razonable RR, Henault M, Paya CV. Stimulation of toll-like receptor 2 with bleomycin results in cellular activation and secretion of pro-inflammatory cytokines and chemokines. Toxicol Appl Pharmacol 2006;210(3):181–9.

44. Ishida Y, Kuninaka Y, Mukaida N, Kondo T. Immune Mechanisms of Pulmonary Fibrosis with Bleomycin. Int J Mol Sci 2023;24(4):3149.

45. Chan AH, Schroder K. Inflammasome signaling and regulation of interleukin-1 family cytokines. J Exp Med 2019;217(1):e20190314.

46. Nakagome K, Dohi M, Okunishi K, Tanaka R, Miyazaki J, Yamamoto K. In vivo IL-10 gene delivery attenuates bleomycin induced pulmonary fibrosis by inhibiting the production and activation of TGF-β in the lung. Thorax 2006;61(10):886–94.

47. Nie YJ, Wu SH, Xuan YH, Yan G. Role of IL-17 family cytokines in the progression of IPF from inflammation to fibrosis. Mil Med Res 2022;9:21.

48. An FGF2-Derived Short Peptide Attenuates Bleomycin-Induced Pulmonary Fibrosis by Inhibiting Collagen Deposition and Epithelial–Mesenchymal Transition via the FGFR/MAPK Signaling Pathway [Internet]. [cited 2025 Apr 1];Available from: https://www.mdpi.com/1422-0067/26/2/517

49. Kohli K, Pillarisetty VG, Kim TS. Key chemokines direct migration of immune cells in solid tumors. Cancer Gene Ther 2022;29(1):10–21.

50. Rosas IO, Richards TJ, Konishi K, Zhang Y, Gibson K, Lokshin AE, et al. MMP1 and MMP7 as Potential Peripheral Blood Biomarkers in Idiopathic Pulmonary Fibrosis. PLOS Med 2008;5(4):e93.
